# Smoldering neuroinflammation in progressive multiple sclerosis: mechanisms, imaging biomarkers, and therapeutic opportunities

**DOI:** 10.3389/fimmu.2026.1901957

**Published:** 2026-09-02

**Authors:** Min Wang, Bo-chi Zhu, Xijing Mao

**Affiliations:** Department of Neurology, The Second Hospital of Jilin University, Changchun, Jilin, China

**Keywords:** chronic active lesions, multiple sclerosis, paramagnetic rim lesions, progressive multiple sclerosis, smoldering neuroinflammation

## Abstract

Progressive multiple sclerosis (MS) remains a major therapeutic challenge because disability often continues to accumulate despite effective control of relapses and new focal inflammatory lesions. This dissociation suggests that progression is driven not only by acute inflammation, but also by a distinct process increasingly termed smoldering neuroinflammation. Recent evidence supports chronic active lesions as an important but non-exclusive pathological substrate of this process. These lesions are characterized by persistent lesion-edge inflammation, slowly expanding tissue injury, iron-laden myeloid cells, astrocyte–immune crosstalk, and incomplete repair. Importantly, this inflammatory activity is not restricted to isolated white matter plaques, but is spatially compartmentalized across the central nervous system, involving interactions among white matter lesions, meninges, cortex, and subcortical regions. Advances in susceptibility-based MRI and PET imaging now allow *in vivo* assessment of imaging-related correlates of this otherwise hidden pathology, including paramagnetic rim lesions (PRLs), slowly expanding lesions (SELs), and TSPO-PET-defined inflammatory activity. Although these biomarkers overlap only partially, they provide complementary insights into lesion composition, structural expansion, and metabolic inflammation. Accumulating studies further suggest that current disease-modifying therapies incompletely control established chronic lesion biology, while other inflammatory-independent or age-related neurodegenerative processes may also contribute to progression. In this review, we discuss the pathological basis, imaging correlates, and therapeutic implications of smoldering neuroinflammation in progressive MS.

## Highlights

- Smoldering neuroinflammation is a major contributor to progression in MS.
- PRLs, SELs, and TSPO-PET reveal chronic active lesions *in vivo*.
- Future therapy must target lesion-edge and CNS-trapped inflammation.

## 1 Introduction

Multiple sclerosis (MS) has traditionally been conceptualized as an autoimmune demyelinating disease driven by episodic inflammatory relapses, blood–brain barrier disruption, and new focal inflammatory lesions (1–3). This model has supported major therapeutic advances, particularly through disease-modifying therapies that reduce relapse frequency and conventional MRI activity. However, it does not fully explain a key clinical problem in progressive MS: disability can continue to accumulate despite apparent control of relapses and new gadolinium-enhancing lesions (4–6). This dissociation suggests that progression is not merely residual relapse-associated inflammation, but may reflect a chronic, compartmentalized pathological process that remains insufficiently captured by conventional clinical and imaging measures.

Recent pathological, molecular, and imaging studies have identified smoldering neuroinflammation and chronic active lesions as central substrates of progressive MS (7–9). These lesions are characterized by persistent lesion-edge inflammation, resident myeloid activation, astrocyte–immune crosstalk, iron accumulation, slow tissue expansion, and incomplete repair (10–12). In parallel, susceptibility-based MRI and PET imaging have enabled *in vivo* assessment of this otherwise hidden pathology through paramagnetic rim lesions (PRLs), slowly expanding lesions (SELs), and TSPO-PET-defined inflammatory activity. However, existing reviews have often discussed chronic active lesions, imaging biomarkers, or progressive MS therapeutics as relatively separate topics. Less attention has been given to how PRLs, SELs, and TSPO-PET provide complementary rather than interchangeable information, and to whether chronic active lesion burden can be linked to treatment response or therapeutic failure.

Therefore, this review aims to integrate three related but incompletely connected areas of the field. First, we summarize the lesion-edge cellular and molecular architecture that sustains smoldering neuroinflammation in progressive MS. Second, we compare PRLs, SELs, and TSPO-PET as complementary imaging windows onto chronic active lesion biology, emphasizing their partial overlap, distinct pathological meanings, and translational limitations (13–15). Third, we discuss how chronic active lesions may inform therapeutic response, treatment resistance, patient stratification, and progression-focused trial design. By linking pathology, multimodal imaging, and therapeutic implications, this review seeks to clarify why smoldering neuroinflammation is not only a mechanistic concept, but also a clinically relevant framework for monitoring and treating progressive MS.

This review includes several categories of evidence: neuropathological and lesion-level molecular studies, susceptibility-based MRI studies, longitudinal SEL analyses, PET studies of innate immune activation, clinical prognostic studies, and treatment-related imaging studies. General reviews and studies of relapse-associated inflammation or conventional DMT mechanisms were used primarily as background. The core conclusions of this review are supported mainly by studies directly addressing chronic active lesions, PRLs, SELs, TSPO-PET-defined inflammatory activity, clinical progression, and treatment-related changes in chronic lesion biomarkers. Throughout the review, we distinguish relatively direct pathological evidence from imaging-related correlates and from more speculative therapeutic implications.

## 2 From relapse-associated inflammation to smoldering progression in MS

The classical inflammatory model of MS is rooted in the biology of relapses: acute focal immune infiltration, formation of new inflammatory lesions, transient blood–brain barrier opening, and periods of partial clinical recovery (16, 17). This model remains highly relevant, particularly for relapsing forms of the disease, and it explains why therapies targeting adaptive immunity can substantially reduce relapse frequency and new MRI activity. Yet it is increasingly clear that this framework cannot fully account for the biology of progressive disability accumulation. In many patients, especially those with secondary progressive or primary progressive MS, worsening neurological impairment continues despite relative suppression of overt relapse-associated inflammation (18–20). This phenomenon indicates that progression reflects not merely a reduction in inflammatory intensity, but a transition to a different inflammatory mode—one that is more chronic, spatially restricted, and closely coupled to irreversible tissue injury.

### 2.1 Why progression persists beyond relapses

One of the central challenges in MS research is explaining why disability progression can continue in patients who appear clinically and radiologically stable by conventional measures. Relapses represent only the most visible component of inflammatory disease activity. By contrast, progressive worsening seems to depend more strongly on persistent low-grade inflammation within established lesions, chronic glial activation, and cumulative neuroaxonal damage. In this setting, tissue injury unfolds over months to years rather than days to weeks, making it less conspicuous clinically but potentially more consequential for long-term neurological decline (21–24). This idea is supported by molecular and imaging evidence showing that chronic lesion activity remains present even in non-acute phases of disease. Jäckle et al. provided a particularly important contribution by demonstrating that slowly expanding lesions in progressive MS have a distinct molecular signature enriched at the lesion rim, with preferential accumulation of resident microglia/macrophage-associated inflammatory programs rather than a pattern dominated by acute peripheral immune infiltration (25). These findings suggest that progression is sustained by lesion-edge processes that persist after the initial lesion-forming event. Absinta et al. extended this concept by revealing a cellular axis involving lymphocytes, microglia, and astrocytes within chronic active lesions, thereby showing that ongoing tissue damage reflects a coordinated inflammatory microenvironment rather than isolated glial activation. Together, these studies argue that the pathological basis of progression lies in a CNS-trapped inflammatory architecture that is at least partially autonomous from classical relapse biology.

Further support for this view comes from biomarker studies linking chronic lesion activity to neuroaxonal injury. Maggi et al. reported that chronic white matter inflammation, as detected by paramagnetic rim pathology on MRI, was associated with increased serum neurofilament light chain (sNfL) levels and greater disease severity in non-acute MS (26). This is an important observation because it bridges lesion-level inflammatory persistence with a systemic biomarker of axonal damage, strengthening the argument that smoldering inflammation is not merely a radiological curiosity but an active driver of neurodegeneration. More recent work has also reinforced the clinical relevance of chronic active lesions at the patient level. Bagnato et al. found that chronic active lesion burden is frequently associated with disease progression and disability accumulation, further supporting the view that these lesions are not incidental findings but rather key indicators of a progression-prone disease state (27).

Taken together, these findings support a conceptual shift: progression beyond relapses should not be interpreted as evidence of inflammatory exhaustion, but as evidence of inflammatory transformation. The dominant biology moves from acute focal lesion formation driven largely by peripheral immune entry to a more compartmentalized process characterized by persistent innate immune activation, chronic lesion-edge inflammation, and cumulative neuroaxonal loss. This distinction has major implications for both disease monitoring and therapeutic strategy, because it suggests that relapse suppression alone is unlikely to fully prevent progression unless the underlying smoldering pathology is also addressed.

### 2.2 Smoldering lesions as the pathological substrate of progression

Chronic active lesions are increasingly recognized as one of the most important pathological substrates linking inflammation to progression in MS. Unlike inactive scars, these lesions remain biologically active at their edges, where inflammatory cells, especially microglia and macrophage-lineage cells, form a rim surrounding a relatively hypocellular demyelinated core (28–30). This rim is not a static boundary. Rather, it reflects a zone of ongoing myelin breakdown, incomplete debris clearance, oxidative stress, and axonal injury. Over time, such lesions can expand slowly, leading to progressive tissue destruction and failure of repair. The concept of the slowly expanding lesion captures this process particularly well and provides a mechanistic explanation for how chronic inflammatory activity can persist long after the acute phase of lesion formation has subsided. The biological significance of these lesions lies not only in their persistence, but also in the type of injury they promote. Chronic active lesions are associated with sustained demyelination, axonal transection, mitochondrial stress, and remyelination failure, all of which contribute to gradual but irreversible functional decline. Because this damage accumulates at the lesion border, it is often poorly captured by conventional MRI measures focused on new lesion counts or gadolinium enhancement (31–33). In this sense, chronic active lesions represent a form of “hidden activity” that is especially relevant to progression independent of relapse activity (PIRA). They provide a pathological framework for understanding why patients may worsen despite the apparent absence of new inflammatory events by standard criteria.

**Figure 1:**
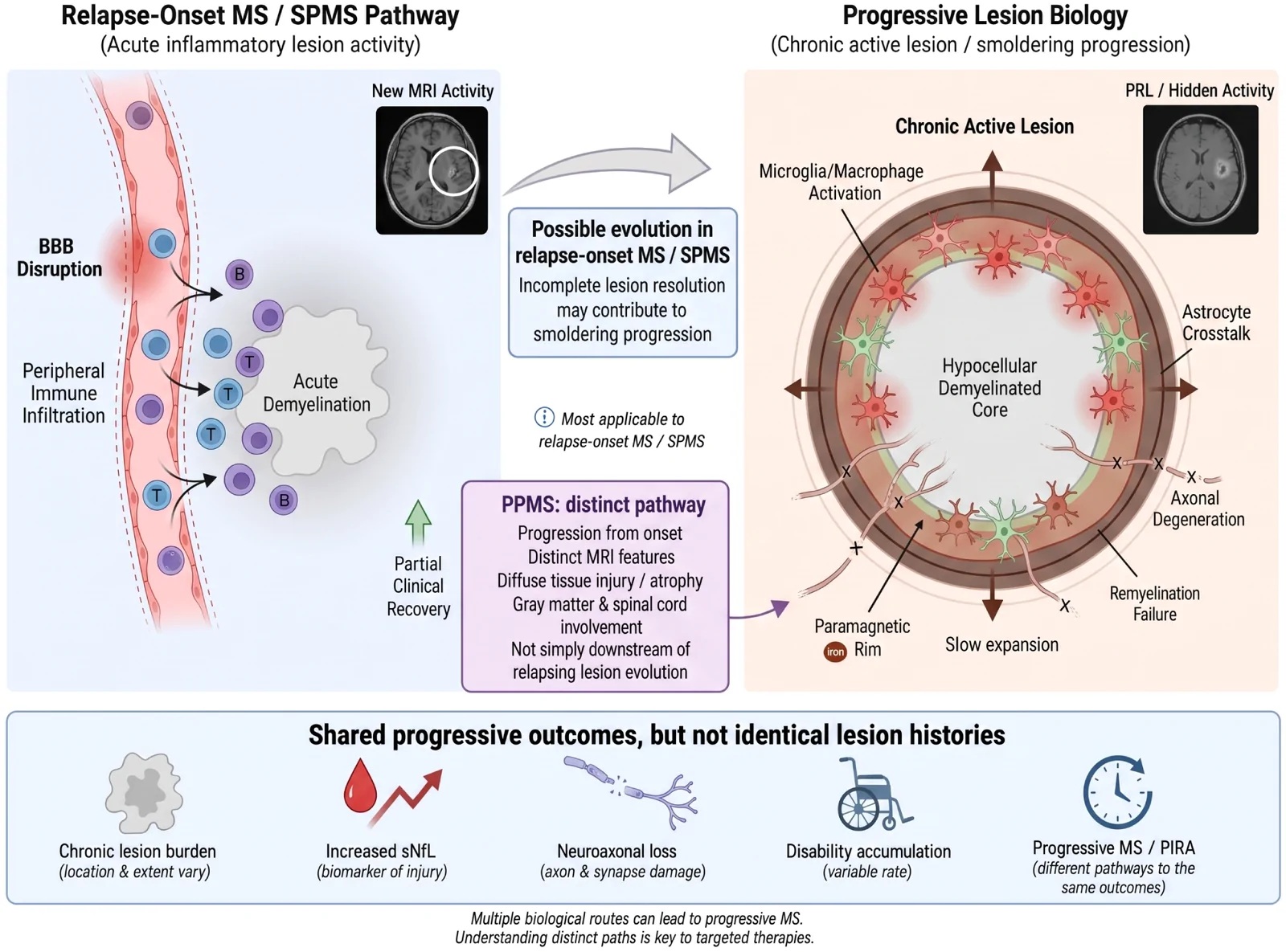
Distinct pathways to progressive MS biology. In relapse-onset MS, particularly secondary progressive MS, some acute inflammatory lesions may incompletely resolve and evolve into chronic active lesions, PRLs, or SELs. In primary progressive MS, progression occurs from onset and should not be represented simply as a downstream stage of relapsing lesion evolution. PPMS may show distinct MRI features, including relatively different patterns of focal lesion activity, spinal cord involvement, gray matter injury, diffuse tissue damage, and atrophy. Chronic active lesions remain relevant, but they represent one component of a broader progressive disease framework.

Imaging and clinical studies increasingly support the role of smoldering lesions as progression-driving structures *in vivo* (34–38). Maggi et al. linked chronic white matter inflammation to higher sNfL levels, implying that these lesions contribute to continuous axonal injury even outside acute relapse periods (26). Bagnato et al. further associated chronic active lesion burden with disability accumulation and clinical progression, reinforcing their importance as markers of disease severity (27). These observations are particularly relevant in the context of PIRA, which is now recognized as a major component of long-term disability accrual in MS. Smoldering lesions offer a biologically plausible substrate for PIRA because they integrate persistent inflammation, ongoing tissue damage, and inadequate repair within a single lesion-centered framework. Accordingly, progressive MS should not be viewed simply as a stage in which inflammation fades and neurodegeneration takes over. Rather, it is better understood as a state in which inflammatory injury becomes chronic, compartmentalized, and structurally embedded within established lesions. Smoldering lesions thus occupy a central position at the interface between inflammation and neurodegeneration: they are inflammatory enough to drive ongoing tissue destruction, yet chronic enough to evade traditional definitions of active disease. Recognizing these lesions as a core pathological substrate of progression is therefore essential for rethinking both how progressive MS is monitored and how next-generation therapies should be designed. Because the concept of chronic active lesions is supported by heterogeneous lines of evidence, it is important to distinguish pathological validation from imaging correlates, clinical prognostic associations, and treatment-response data. These evidence types do not carry the same inferential weight. Neuropathological and lesion-level molecular studies provide relatively direct validation of chronic active lesion biology, whereas longitudinal MRI and PET studies provide *in vivo* correlates of lesion persistence, expansion, and inflammatory metabolism (39, 40). Clinical outcome studies further support the prognostic relevance of chronic active lesions, but causal links to disability accumulation and therapeutic response remain incompletely established. **Table 1** summarizes this evidence hierarchy and distinguishes relatively stable conclusions from more speculative or emerging interpretations. **Figure 1** illustrates the transition from acute relapse-associated inflammation to smoldering progression in multiple sclerosis. It highlights chronic active lesions as the pathological substrate linking CNS-compartmentalized inflammation to persistent tissue injury, neuroaxonal loss, and progression independent of relapse activity.

**Table 1:** Evidence hierarchy supporting chronic active lesions as substrates of smoldering progression in multiple sclerosis.

Evidence category	Representative studies/evidence	Main conclusion supported	Strength of conclusion	Remaining uncertainty	Representative PMID
Neuropathological validation	Post-mortem and lesion-level studies showing hypocellular demyelinated cores, inflammatory rims, and persistent lesion-edge activity	Chronic active lesions are real pathological entities rather than imaging artifacts	Relatively stable	Degree of heterogeneity among lesion subtypes remains incompletely defined	32577755
Lesion-edge molecular profiling	Jäckle et al. identified molecular signatures of slowly expanding lesions enriched at lesion borders	Slowly expanding lesions have distinct inflammatory programs at the rim	Relatively stable	Whether all SELs represent the same pathological stage remains uncertain	32577755
Multicellular lesion biology	Absinta et al. described a lymphocyte–microglia–astrocyte axis in chronic active MS lesions	Chronic lesion persistence reflects a multicellular inflammatory niche	Relatively stable	The relative contribution of each cell type may vary across patients and disease stages	34497421
Iron-related pathological validation	Hofmann et al. linked myeloid iron uptake pathways with paramagnetic rim formation	Iron-laden myeloid cells provide a pathological basis for PRLs	Relatively stable	Iron accumulation may not capture all forms of chronic lesion activity	37715818
Longitudinal PRL imaging	Dal-Bianco et al. and Reeves et al. showed long-term evolution and prognostic relevance of iron rim lesions	PRLs can persist over time and are associated with more aggressive disease evolution	Moderately stable	Imaging protocols and field strength influence PRL detection	33484118; 40357663
Longitudinal SEL imaging	Elliott et al. and Calvi et al. linked slowly expanding lesions with chronic lesion expansion and disability in SPMS	SELs capture structural lesion expansion over time	Moderately stable	SEL definitions and segmentation pipelines vary across studies	35277438
PRL–SEL correspondence	Elliott et al. showed only partial lesion-level overlap between PRLs and SELs	PRLs and SELs are complementary rather than interchangeable biomarkers	Relatively stable	The biological meaning of PRL-only and SEL-only lesions requires further validation	37036134
PET evidence	Hamzaoui et al. and Polvinen et al. showed TSPO-PET-detectable chronic inflammatory activity associated with progression	PET provides evidence that chronic lesions can remain metabolically inflammatory *in vivo*	Moderately stable	TSPO specificity, accessibility, and clinical standardization remain limitations	37039158
Clinical prognostic studies	Studies linking PRL/SEL/chronic lesion burden with sNfL, atrophy, disability, or progression	Chronic active lesion burden is clinically relevant and may predict worse outcomes	Moderately stable	Association does not fully prove causality	34088875; 40357663
Treatment-response studies	Maggi et al. showed B-cell depletion does not resolve chronic active lesions; Zinger et al. reported partial reduction of inflammation with dimethyl fumarate	Current DMTs may incompletely control chronic lesion biology	Emerging/partly speculative	Treatment-response evidence remains limited and requires prospective validation	37437310; 35046083
Trial enrichment and therapeutic targeting	Imaging biomarkers proposed for stratification and progression-focused endpoints	PRLs, SELs, and TSPO-PET may help select patients and monitor lesion-directed therapies	Speculative/emerging	Clinical utility as validated trial endpoints remains to be proven	37349108

### 2.3 Chronic active lesions are important but not exclusive substrates of progression

Although chronic active lesions provide a valuable lesion-level framework for understanding smoldering progression, they should not be viewed as the only mechanism of progressive MS. Disability accumulation may also reflect diffuse injury in normal-appearing white matter and gray matter, cortical demyelination, meningeal inflammation, mitochondrial dysfunction, impaired remyelination, brain and spinal cord atrophy, synaptic and network degeneration, and age-related decline in repair capacity (41–43). These processes may interact with chronic lesion activity, but they are not necessarily reducible to PRLs, SELs, or TSPO-PET-defined inflammatory lesions. Therefore, CAL/PRL biology is best interpreted as one important component of a broader progressive disease network.

### 2.4 Applicability to SPMS and PPMS

The concept that acute inflammatory lesions may incompletely resolve and later evolve into chronic active lesions is most directly applicable to relapse-onset MS and secondary progressive MS (44, 45). In this setting, a temporal relationship between earlier focal inflammatory lesion formation and later chronic lesion activity is biologically plausible. However, this framework should not be generalized uncritically to primary progressive MS. PPMS is characterized by progression from disease onset and may differ from RRMS and SPMS in clinical presentation, lesion distribution, spinal cord involvement, diffuse tissue injury, and MRI activity patterns. Therefore, chronic active lesions and PRLs in PPMS should be interpreted as part of progressive MS biology, but not necessarily as the endpoint of a prior relapsing inflammatory lesion sequence. This distinction is important because PPMS remains less extensively studied as an independent group in chronic active lesion and PRL research. Future studies should stratify analyses by clinical phenotype, especially RRMS, SPMS, and PPMS, rather than assuming that lesion evolution follows the same temporal pathway across all forms of MS.

## 3 Cellular and molecular architecture of chronic active lesions

At the pathological level, chronic active lesions provide some of the most direct evidence for smoldering neuroinflammation in progressive multiple sclerosis (MS). Far from being inert remnants of earlier inflammatory attacks, these lesions represent biologically active microenvironments in which inflammatory, metabolic, and degenerative processes remain engaged over prolonged periods. Their defining feature is not simply persistent demyelination, but the presence of a chronically inflamed lesion edge that sustains low-grade tissue injury long after the acute phase of lesion formation has subsided (46–48). Molecular profiling studies have shown that this lesion border is enriched for immune and glial programs that differ substantially from those observed in inactive lesion cores, indicating that chronic active lesions are organized as spatially specialized niches rather than uniform scars. A central concept emerging from recent work is that the lesion edge functions as a dynamic interface between inflammation and neurodegeneration. At this border, myeloid cells, astrocytes, lymphocytes, and iron-handling pathways interact to maintain a state of persistent but incomplete inflammatory resolution (49–52). In this context, lesion expansion reflects not merely passive structural instability, but the continued operation of cellular programs linked to myelin breakdown, impaired debris clearance, oxidative injury, and failed repair. Absinta et al. highlighted this biology through the description of a lymphocyte–microglia–astrocyte axis within chronic active lesions, whereas Jäckle et al. provided complementary molecular evidence showing that slowly expanding lesions are marked by lesion-edge inflammatory specialization. More recent work has further linked this biology to iron metabolism and the formation of paramagnetic rims, reinforcing the view that chronic active lesions are best understood as metabolically and immunologically active compartments of ongoing injury. **Table 2** summarizes the key cellular and molecular features of chronic active lesions in progressive multiple sclerosis, highlighting the lesion-edge microenvironment, persistent myeloid activation, astrocyte–immune crosstalk, iron metabolism, and failed repair processes that sustain smoldering neuroinflammation.

**Table 2:** Cellular and molecular architecture of chronic active lesions in progressive multiple sclerosis.

Feature	Main pathological/cellular components	Biological significance	Clinical/review relevance	Representative PMID
Lesion core	Hypocellular, chronically demyelinated, axon-poor tissue	Reflects established irreversible tissue damage rather than ongoing active injury	Explains why the lesion center is less informative than the rim for understanding progression	32577755
Lesion edge (rim)	Activated microglia/macrophage-lineage cells enriched at lesion border	Major site of persistent low-grade inflammation and slow lesion expansion	Supports the concept that chronic active lesions are border-driven rather than inactive scars	32577755
Myeloid activation	Phagocytosis, myelin debris uptake, inflammatory mediator release, oxidative stress	Sustains smoldering injury and prevents full lesion resolution	Positions microglia/myeloid cells as central effectors of chronic lesion persistence	34497421
Astrocyte–immune crosstalk	Astrocytes interacting with microglia and lymphocytes	Amplifies inflammatory signaling, destabilizes tissue homeostasis, and inhibits repair	Explains why lesion persistence reflects a multicellular niche rather than a single-cell process	34497421
Lymphocyte–microglia–astrocyte axis	Coordinated interaction among lymphocytes, microglia/macrophages, and astrocytes	Stabilizes chronic lesion-edge inflammation	Provides a mechanistic framework for lesion compartmentalization and persistence	34497421
Iron handling at lesion rim	Iron-laden myeloid cells, heme/scavenger-related programs	Links chronic inflammation to oxidative stress and PRL formation	Connects lesion biology to MRI-visible paramagnetic rims	37715818
Oxidative/metabolic stress	Reactive oxygen species, mitochondrial dysfunction, impaired redox balance	Promotes axonal stress and tissue degeneration	Helps explain why chronic lesions contribute to neurodegeneration	37715818
Myelin debris accumulation	Incomplete debris clearance at lesion border	Maintains non-resolving inflammatory state and impairs remyelination	Suggests lesion persistence reflects failed resolution, not simple inflammatory quiescence	34497421
Failed remyelination/repair	Inhibited regenerative microenvironment	Prevents restoration of function and promotes chronic damage accumulation	Important for linking chronic inflammation to irreversible progression	37276054

### 3.1 The lesion edge as a biologically active niche

One of the most important conceptual advances in the field has been the recognition that the biologically relevant compartment of a chronic active lesion is its edge rather than its center. The lesion core is typically characterized by severe demyelination, axonal loss, and relative hypocellularity, reflecting tissue that has already undergone substantial destruction (53–57). By contrast, the lesion rim remains biologically active and appears to host the cellular processes that drive continued lesion expansion. Jäckle et al. showed that slowly expanding lesions in progressive MS display a distinct molecular signature concentrated at the lesion border, with enrichment of microglia/macrophage-related inflammatory pathways that support ongoing myelin breakdown and axonal injury (58, 59). This finding helped redefine chronic active lesions as border-driven pathological entities and provided a molecular basis for the long-held neuropathological observation that lesion edges are the sites of persistent activity. The importance of the lesion edge lies in its role as a transition zone where damaged but not yet completely destroyed tissue remains exposed to chronic inflammatory stress. This border region likely represents a site of repeated failure of lesion resolution, where persistent innate immune activation, incomplete clearance of myelin debris, and ongoing metabolic stress prevent the shift toward true tissue quiescence. Rather than resolving into stable scars, these lesions maintain a slow-burning state in which expansion occurs over time through continuing damage at the margin. This spatial organization has major implications for both pathology and imaging, because it means that biologically meaningful disease activity may persist even when conventional indicators of acute inflammation are absent.

### 3.2 Microglia and myeloid cells as effectors of smoldering injury

Among the cellular players implicated in chronic active lesions, microglia and other myeloid-lineage cells appear to be the principal executors of smoldering tissue injury (60–62). These cells accumulate at the lesion rim and display transcriptional and functional features consistent with chronic activation, phagocytic engagement, iron handling, and oxidative stress. Their role is likely multifaceted: they participate in myelin debris uptake, secrete inflammatory mediators, regulate local redox balance, and shape the extracellular environment in ways that may perpetuate both tissue destruction and failed repair. Absinta et al. demonstrated substantial glial and immune-cell diversity at the chronically inflamed lesion edge, underscoring that lesion-associated myeloid cells are not a homogeneous population but rather a spectrum of activated states linked to chronic lesion pathology.

A particularly important development came from Hofmann et al., who connected myeloid biology to iron metabolism and paramagnetic rim formation. Their work showed that sustained inflammatory activity at chronic lesion edges is associated with specific iron uptake pathways in myeloid cells, including programs involving scavenger and heme-handling molecules (63). This suggests that iron accumulation in chronic active lesions is not a passive byproduct of tissue injury alone, but is closely tied to the phenotype of lesion-edge myeloid cells. In practical terms, this means that the paramagnetic rim seen on iron-sensitive MRI is a readout of a defined inflammatory-metabolic state rather than simply a structural imaging artifact. These observations strengthen the idea that myeloid cells sit at the center of lesion persistence by integrating phagocytosis, oxidative injury, and iron-driven inflammatory stress. The pathological consequences of this sustained myeloid activation are likely broad. In addition to ongoing myelin destruction, chronically activated myeloid cells may contribute to mitochondrial dysfunction, axonal stress, and inhibition of remyelination through persistent release of inflammatory and toxic mediators. Abnormal processing of myelin debris may further reinforce lesion chronicity by maintaining a non-resolving inflammatory state. Thus, lesion-edge myeloid cells should not be viewed simply as markers of smoldering pathology; they are likely central drivers of its continuation.

### 3.3 Astrocyte–immune crosstalk in lesion persistence

Although microglia and macrophage-lineage cells occupy a prominent position in chronic active lesion biology, astrocytes are increasingly recognized as equally important participants in sustaining lesion persistence. Traditionally considered reactive but secondary responders, astrocytes are now understood to contribute actively to inflammatory organization, barrier regulation, metabolic support failure, and inhibition of repair. Absinta et al. provided particularly strong evidence for this view by identifying an integrated lymphocyte–microglia–astrocyte axis within chronic active MS lesions (10). Their data suggest that astrocytes are not merely responding to pre-existing injury, but are embedded within a multi-cellular inflammatory network that helps stabilize the chronically active lesion edge. Astrocytes may promote lesion persistence in several ways. They can amplify local inflammatory signaling, alter the extracellular milieu, and modulate the behavior of both infiltrating lymphocytes and resident myeloid cells. They may also contribute to repair failure by generating an environment that is unfavorable to oligodendrocyte progenitor differentiation and effective remyelination. In this sense, astrocytes occupy a strategic position at the interface between inflammation and regeneration: they can either facilitate tissue recovery or lock lesions into a non-resolving state (12, 64–67). Within chronic active lesions, the balance appears to shift toward the latter. This helps explain why lesion persistence is not simply a matter of continued immune-cell presence, but rather the product of sustained cross-talk among multiple CNS-resident and immune-derived cell types. This broader cellular framework is important because it moves the field away from overly reductionist interpretations of chronic lesion pathology. Smoldering injury is not maintained by one dominant cell type acting in isolation. Instead, it emerges from a coordinated lesion-edge ecosystem in which astrocytes, myeloid cells, and lymphocytes reinforce one another’s pathological programs. Such a model is likely to be more useful than single-cell explanations when considering why these lesions persist over time and why they remain difficult to treat.

### 3.4 Iron-laden rims: pathological correlate, bystander, or active mediator?

**Figure 2:**
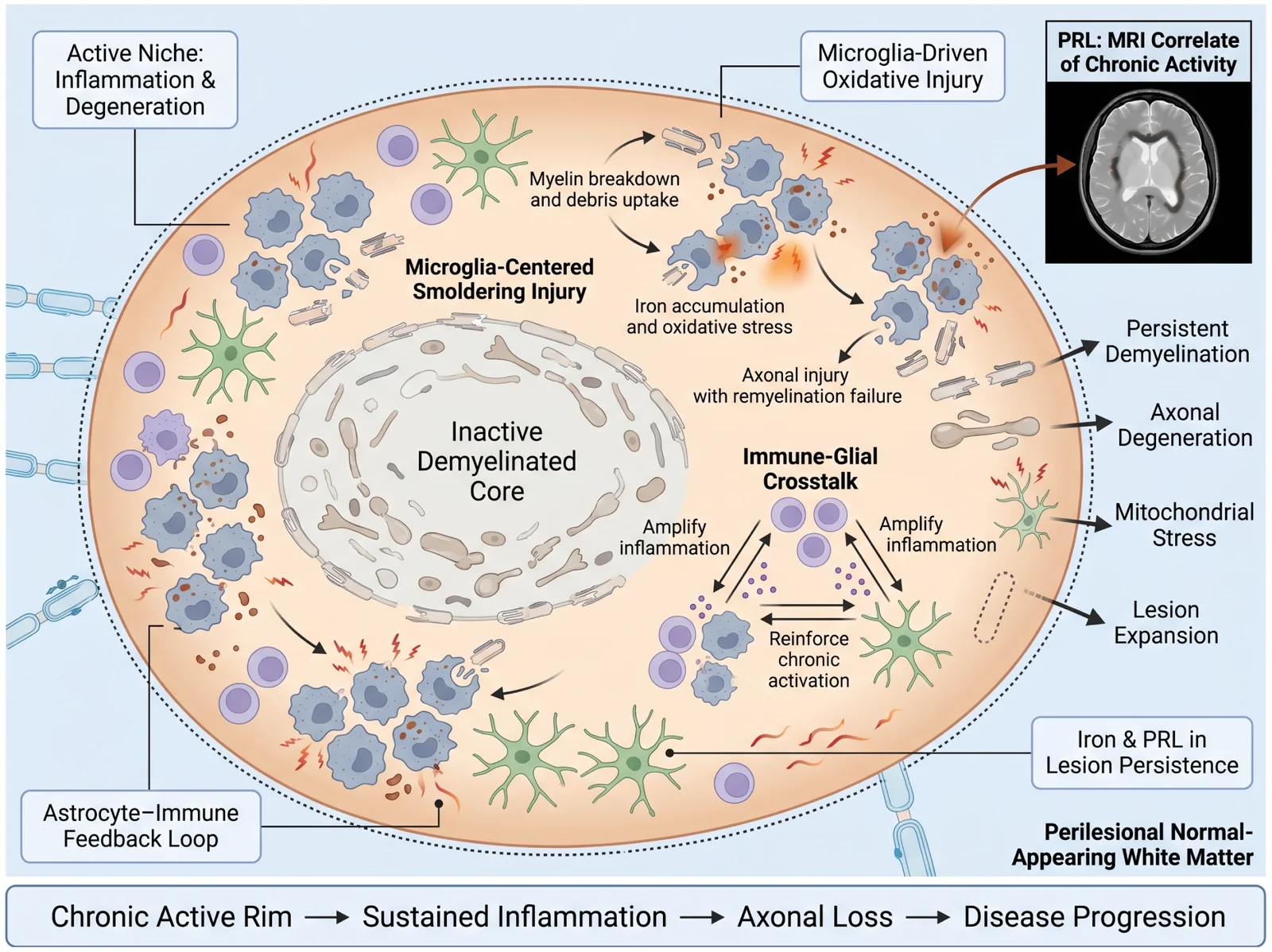
Cellular and molecular architecture of chronic active lesions in progressive multiple sclerosis. Chronic active lesions contain an inactive demyelinated core and a biologically active lesion edge enriched with microglia/macrophages, astrocytes, lymphocytes, iron-handling pathways, oxidative stress, and myelin debris. This organized inflammatory rim sustains persistent tissue injury, axonal damage, remyelination failure, and slow lesion expansion, while also providing the pathological basis for the MRI-visible paramagnetic rim lesion.

The study of paramagnetic rim lesions (PRLs) has provided a major bridge between lesion pathology and *in vivo* imaging. PRLs are increasingly interpreted as the MRI-visible correlate of chronic active lesions, and their defining paramagnetic edge is thought to arise from iron-laden inflammatory cells at the lesion border (68–72). PRLs are increasingly interpreted as susceptibility-based imaging correlates of chronic active lesion biology, and their defining paramagnetic edge is commonly attributed to iron-laden inflammatory cells at the lesion border. However, the biological meaning of iron accumulation at lesion rims requires cautious interpretation. Current evidence supports an association among iron handling, lesion-edge myeloid activity, oxidative stress, and tissue injury, but it does not definitively establish iron itself as a direct causal driver of lesion persistence or expansion (73, 74). Hofmann et al. offered strong mechanistic support for this interpretation by showing that iron uptake and handling pathways are specifically linked to lesion-edge myeloid cells in MS (63). Their findings suggest that iron accumulation reflects a biologically meaningful component of chronic lesion activity, tied to phagocytic function, oxidative stress, and inflammatory persistence. Importantly, the rim should not be seen merely as an imaging signature of past tissue damage. An alternative interpretation is that iron accumulation may partly represent a downstream footprint of prior tissue destruction rather than an active pathogenic trigger. During chronic demyelination, iron may be released from damaged oligodendrocytes, myelin, or other cellular components and subsequently taken up by phagocytic microglia/macrophages engaged in debris clearance (75–77). In this scenario, iron-laden rims would mark previous myelin breakdown, impaired debris processing, and sustained phagocytic activity, without necessarily proving that iron accumulation independently drives lesion expansion. Therefore, iron should be interpreted as a biologically informative correlate of chronic lesion activity, but not automatically as a causative mechanism. Choi et al. showed using 7-Tesla MRI that PRLs are associated with abnormalities extending beyond iron sensitivity alone, including blood–brain barrier alterations, demyelination, and neurodegeneration-related imaging features (78). These observations support the interpretation that PRLs identify lesions with ongoing structural and physiological abnormalities, although they do not prove that iron accumulation itself is the initiating or sustaining cause of this pathology. In other words, the paramagnetic rim is best understood as a window into a lesion state characterized by chronic inflammation and continued tissue vulnerability. This framing has major implications for the interpretation of lesion heterogeneity in progressive MS. Iron-laden rims identify lesions in which susceptibility changes, myeloid activity, and tissue injury are spatially organized (79, 80). Whether iron is a passive marker of prior damage, a contributor to oxidative stress, or an active mediator of continued expansion likely varies across lesions and remains incompletely resolved. Because these lesions connect a specific myeloid phenotype, a measurable imaging signature, and a plausible mechanism of ongoing tissue destruction, they are especially attractive as biomarkers of progression-driving pathology. More broadly, they illustrate how the cellular and molecular architecture of chronic active lesions can be translated into clinically accessible markers of disease biology. **Figure 2** illustrates chronic active lesions as border-driven inflammatory niches rather than inactive scars in progressive multiple sclerosis. It highlights how lesion-edge interactions among myeloid cells, astrocytes, lymphocytes, and iron-related pathways sustain persistent injury and generate the pathological basis of paramagnetic rim lesions.

## 4 Compartmentalized inflammation beyond white matter lesions: meningeal, cortical, and subcortical interactions

A key question in the study of smoldering neuroinflammation is whether chronic lesion activity is confined to white matter plaques or instead reflects a broader spatially organized inflammatory network within the CNS. Emerging pathological and imaging evidence supports a more spatially distributed interpretation. In progressive MS, inflammation may become compartmentalized across anatomical regions, involving not only established white matter lesions but also the meninges, cortex, and subcortical or juxtacortical white matter (9, 15, 81, 82). This expanded view is important because it suggests that progressive disease cannot be fully explained by isolated lesion-centered pathology. Rather, lesion activity may be embedded within a larger tissue context in which inflammatory processes propagate or reinforce one another across adjacent compartments. This concept is especially relevant for understanding why progression often continues despite control of acute peripheral inflammation. If chronic inflammatory activity is sustained within CNS-restricted niches, then pathology may become partially uncoupled from the classic relapse model based on recurrent waves of peripheral immune entry. Compartmentalization would therefore help explain both the persistence of disease worsening and the limited impact of therapies that are highly effective against acute inflammatory lesion formation but less effective against progression. Absinta et al. already hinted at such lesion-intrinsic compartmentalization through the description of organized immune–glial interactions at chronic lesion edges. More recent studies of meningeal and subcortical pathology extend this logic further by showing that the relevant inflammatory architecture may span multiple tissue planes.

### 4.1 Meningeal lymphoid inflammation in progressive MS

Meningeal inflammation has emerged as one of the most important non-parenchymal components of progressive MS pathology. Ahmed et al. demonstrated that the accumulation of meningeal lymphocytes correlates with white matter lesion activity in progressive MS and supports the involvement of meningeal immune processes in subpial cortical injury (83). Importantly, this evidence supports a spatial association between meningeal inflammation and parenchymal lesion activity, but it does not by itself prove a direct one-way transmission of inflammation from the meninges to white matter lesions. This is a significant finding because it challenges the notion that meningeal inflammation is relevant only to cortical pathology. Instead, it suggests that meningeal immune-cell aggregates may be linked more broadly to lesion activity in adjacent CNS compartments. The pathological importance of meningeal lymphoid inflammation likely lies in both its anatomical position and its chronicity. Located at the interface between cerebrospinal fluid spaces and cortical surfaces, meningeal immune infiltrates are well positioned to influence superficial cortical tissue and nearby white matter through diffusible mediators and sustained local inflammatory signaling (84–88). In progressive MS, where overt blood–brain barrier disruption is less prominent than in acute relapsing disease, meningeal niches may provide a relatively sheltered compartment in which inflammatory interactions persist over time. This could help explain why cortical injury and white matter lesion activity remain linked even in later disease stages.

### 4.2 Spatial associations among meningeal, cortical, and white matter inflammation

The idea of compartmentalized inflammation becomes even more compelling when considering evidence that pathology across the meninges, cortex, and subcortical white matter may be coordinated rather than isolated (89–92). Okutan et al. addressed this issue by asking whether lesions and inflammatory activity in the sub/juxtacortical white matter reflect cortical and meningeal pathologies in progressive MS (93). Their findings support a broader spatial association: subcortical plaque activity and inflammatory infiltrates were related to cortical and meningeal inflammatory changes (84, 94–96). These data suggest that lesion activity in progressive MS may be embedded within a regional inflammatory context rather than representing a purely isolated white matter event. However, this relationship should be interpreted primarily as spatial correlation unless supported by longitudinal or spatially registered evidence showing directional propagation. This shift from a single-lesion narrative to a regional network model has major conceptual implications. It suggests that chronic lesion activity in progressive MS may be maintained not only by the cellular programs within a given lesion, but also by ongoing cross-talk between anatomically adjacent compartments. Sub/juxtacortical lesions may therefore serve as accessible surrogates for deeper pathological processes involving cortical and meningeal inflammation. Such a framework is especially attractive because it offers a plausible bridge between white matter pathology, cortical dysfunction, and the cognitive and progressive features of the disease. It also aligns well with the broader concept of CNS compartmentalization, in which inflammation becomes spatially organized and partially self-sustaining within the CNS.

### 4.3 Why this matters for progression and therapy resistance

**Figure 3:**
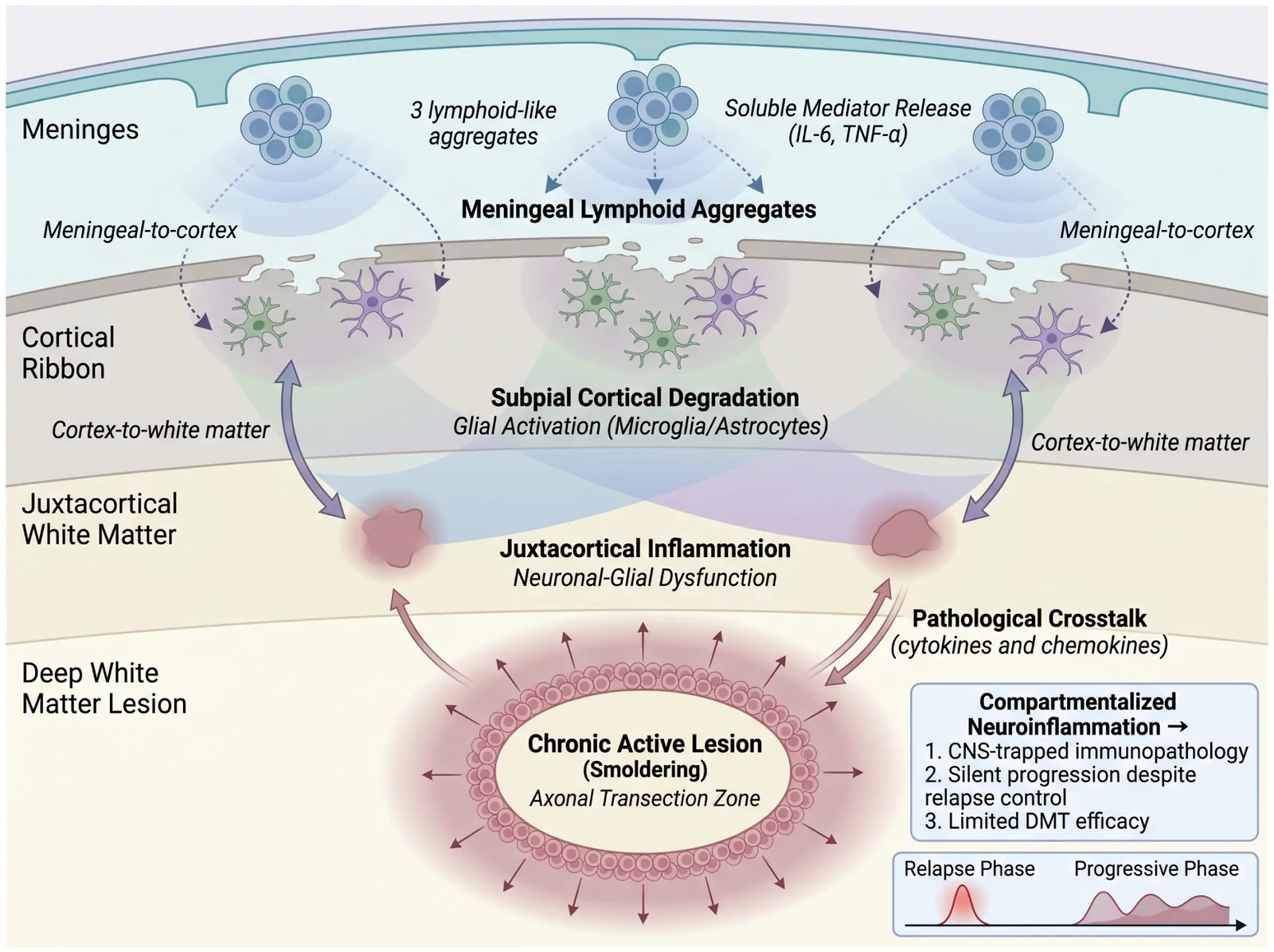
Compartmentalized inflammation beyond white matter lesions in progressive multiple sclerosis. Smoldering neuroinflammation in progressive MS extends across the meninges, cortex, subcortical/juxtacortical white matter, and chronic active white matter lesions. These interconnected CNS-restricted inflammatory niches form a meningeal–cortical–white matter continuum that sustains persistent tissue injury, progression independent of relapse activity, and incomplete therapeutic control despite apparent suppression of acute focal inflammation.

Recognizing that smoldering inflammation may extend beyond white matter lesions is important not only for pathology, but also for clinical interpretation and therapeutic design. If progressive MS is sustained by compartmentalized inflammatory networks involving meninges, cortex, and lesion-bearing white matter, then the biological target of therapy is broader and more complex than the suppression of new focal inflammatory lesions (93, 97, 98). In such a context, therapies that effectively reduce acute peripheral immune activity may leave CNS-restricted inflammatory niches relatively intact, allowing chronic lesion activity and tissue degeneration to continue. This idea is consistent with the broader observation that progression can persist despite successful suppression of relapses and new gadolinium-enhancing lesions. The concept of compartmentalization also helps explain why progressive MS often appears biologically active even when standard clinical and MRI metrics suggest relative stability (99, 100). A patient may have no obvious new lesions, yet still harbor ongoing inflammatory interactions across the meninges, cortex, and chronic lesion edges. This hidden activity is likely to be particularly relevant to progression independent of relapse activity and may underlie the incomplete efficacy of current DMTs in preventing long-term disability accumulation. From this perspective, understanding trans-compartment inflammatory organization is not merely an academic refinement; it is a prerequisite for designing therapies capable of addressing the forms of inflammation that remain operational in progressive disease. **Figure 3** illustrates a putative spatial relationship among meningeal, cortical, subcortical, and white matter inflammatory compartments in progressive multiple sclerosis. It highlights how these anatomically related regions may show coordinated inflammatory activity, while emphasizing that direct pathological transmission and mutually reinforcing mechanisms remain to be validated.

### 4.4 Tissue injury beyond focal lesions: leukocortical lesions, NAWM damage, and gray matter degeneration

Progressive MS pathology is not restricted to focal chronic active white matter lesions. Leukocortical lesions, which involve both cortical gray matter and adjacent subcortical white matter, represent an important interface between focal lesion pathology and broader cortical injury (101, 102). These lesions may be particularly relevant in the setting of meningeal inflammation, because inflammatory activity near the cortical surface may coexist with cortical demyelination, subcortical plaque activity, and regional neurodegeneration. Therefore, leukocortical lesions provide a useful anatomical bridge between the meningeal–cortical inflammatory compartment and focal white matter lesion biology. In addition, normal-appearing white matter and gray matter can show diffuse abnormalities despite the absence of conventional focal lesions. These changes may include axonal injury, microglial activation, mitochondrial dysfunction, synaptic loss, impaired remyelination, and tissue atrophy (103–105). Such diffuse injury may contribute to disability accumulation independently of, or in parallel with, PRLs and SELs. Therefore, PRLs, SELs, and TSPO-PET-positive lesions should be interpreted as important but incomplete windows onto progressive pathology. A more integrated framework should consider how focal chronic lesion activity interacts with diffuse white matter damage, gray matter degeneration, cortical atrophy, and loss of neural network integrity.

## 5 Imaging the invisible: MRI and PET biomarkers of smoldering neuroinflammation

At the *in vivo* imaging level, smoldering neuroinflammation is inferred through biomarkers that capture different and only partially overlapping aspects of chronic lesion activity. New gadolinium-enhancing lesions and relapse counts are useful for identifying acute focal inflammation, but they are poorly suited to detecting chronic lesion-edge activity that persists over months or years. Recent advances in susceptibility-based MRI, longitudinal lesion tracking, and positron emission tomography (PET) have therefore been transformative (106–108). These methods now make it possible to visualize, at least indirectly, the pathological substrate of chronic active lesions *in vivo*. In particular, paramagnetic rim lesions (PRLs), slowly expanding lesions (SELs), and TSPO-PET-defined chronic inflammatory lesions have emerged as leading biomarkers of smoldering pathology in progressive multiple sclerosis (MS) (109–112). Importantly, however, these markers should not be treated as fully interchangeable. Rather, they represent overlapping but distinct windows onto a shared pathological spectrum of chronic lesion activity. Elliott et al. provided especially important evidence for this view by showing that PRLs and SELs overlap only partially at the lesion level, indicating that each biomarker captures somewhat different aspects of chronic active lesion biology. This distinction matters conceptually and clinically. If PRLs, SELs, and TSPO-PET all index chronic activity but emphasize different biological properties—such as iron-laden lesion rims, slow lesion expansion, or persistent innate immune metabolic activation—then no single modality can be assumed to exhaustively define chronic active pathology (113–115). Instead, the field is moving toward a multimodal framework in which lesion behavior, composition, and inflammatory state are interpreted together. This is especially relevant in progressive MS, where conventional MRI may underestimate biologically meaningful ongoing disease activity. A strength of the current literature is that it increasingly links these imaging features not only to pathology, but also to disability accumulation, brain atrophy, and clinical worsening. **Table 3** compares the main *in vivo* imaging biomarkers—PRLs, SELs, and TSPO-PET—used to detect smoldering lesion activity, emphasizing their complementary roles in capturing lesion-edge inflammation, slow structural expansion, and metabolically active chronic inflammation. In this section, PRLs, SELs, and TSPO-PET-defined inflammatory lesions are discussed as imaging-related markers rather than direct pathological equivalents of chronic active lesions. PRLs primarily reflect susceptibility changes associated with iron-laden lesion rims, SELs capture longitudinal structural expansion, and TSPO-PET reflects metabolically active innate immune inflammation. Therefore, these markers should be interpreted as complementary but non-interchangeable windows onto chronic active lesion biology.

**Table 3:** *In vivo* imaging biomarkers of smoldering neuroinflammation in progressive multiple sclerosis.

Biomarker	What it detects	Main biological/pathological correlate	Strengths	Limitations	Clinical significance	Representative PMID
Paramagnetic rim lesion (PRL)	Susceptibility-based MRI/QSM rim signal around white matter lesion	Iron-laden inflammatory cells at lesion edge; chronic lesion-edge activity	Strong pathological specificity for chronic active lesion rim; linked to tissue damage and prognosis	Depends on imaging technique and field strength; not all chronic active lesions are PRL-positive	Marker of persistent lesion-edge inflammation, worse tissue damage, and higher progression risk	33484118
Slowly expanding lesion (SEL)	Longitudinal structural enlargement of an established lesion on MRI	Ongoing slow lesion growth driven by chronic border activity	Captures dynamic structural progression over time; relevant in SPMS	Requires longitudinal imaging and dedicated analysis pipelines	Useful marker of chronic lesion expansion and progression-related tissue damage	35277438
TSPO-PET-defined chronic active lesion	Increased PET signal reflecting activated innate immune metabolism	Persistent microglial/macrophage activation and metabolically active chronic inflammation	More direct readout of inflammatory metabolism than MRI alone	Less accessible, more expensive, limited routine clinical use	Strong evidence that chronic lesions remain biologically active and progression-relevant	37039158
PRL + SEL overlap	Lesions positive for both rim signal and slow expansion	Severe chronic active lesion phenotype	Improves lesion phenotyping; identifies potentially more destructive lesions	Partial overlap only; cannot treat the two markers as interchangeable	Suggests that multimodal lesion characterization may better stratify disease severity	37036134
QSM-defined PRL	Rim-positive lesions with higher pathological specificity	More severe myelin damage and stronger clinicoradiologic relevance	Better lesion characterization than phase imaging	Requires optimized susceptibility imaging	Supports more precise identification of chronic active lesions	35262241
3T rim lesion detection	PRL-like lesions detected on 3T MRI	Feasible lower-field detection of chronic active rims	More clinically translatable than ultra-high-field imaging	Lower sensitivity/specificity compared with higher-field methods	Encourages broader clinical adoption of chronic lesion imaging	37907156
Broad rim lesion	Lesion with broader inflammatory rim pattern	Potentially more aggressive chronic active lesion subtype	May identify lesions associated with faster progression	Still emerging; needs further validation	Supports the idea that chronic active lesions are biologically heterogeneous	40301560
Conventional gadolinium-enhancing lesion	Acute BBB leakage and focal inflammatory activity	Acute peripheral-entry-driven lesion formation	Useful for acute inflammatory monitoring	Poorly captures chronic lesion-edge activity and smoldering pathology	Highlights why conventional MRI alone underestimates progression-driving biology	37036134

### 5.1 Paramagnetic rim lesions: pathological meaning and longitudinal evolution

PRLs are currently among the most intensively studied imaging correlates of chronic active lesions. They are identified on susceptibility-sensitive MRI as white matter lesions bordered by a paramagnetic rim, which is generally interpreted as reflecting iron-laden inflammatory cells at the lesion edge. Their significance lies in the fact that they appear to mark lesions in which inflammatory activity remains spatially organized and biologically persistent rather than fully resolved. Dal-Bianco et al. provided a foundational longitudinal study showing that multiple sclerosis iron rim lesions evolve over many years after their initial formation, supporting the idea that they represent durable chronic active lesions rather than transient remnants of earlier inflammation (116). This observation is central to the concept of smoldering lesion biology: the rim is not simply a sign of prior injury, but evidence of a lesion state that remains active over extended periods. Subsequent studies have strengthened the clinical relevance of PRLs. Marcille et al. showed that rim-positive lesions identified on quantitative susceptibility mapping (QSM) are associated with both clinical disability and more severe tissue damage, supporting the idea that PRLs mark lesions of greater pathological significance (117). More recent longitudinal work by Reeves et al. further demonstrated that resolution of existing PRLs and the absence of new PRLs are associated with better clinical outcomes, indicating that PRLs may serve not only as static prognostic features but also as dynamic markers of disease evolution (118). In a separate long-term study, Reeves and colleagues also reported that PRLs predict higher future relapse rates and greater long-term disability progression, reinforcing the view that these lesions identify an aggressive ongoing inflammatory disease state. Together, these studies support interpreting PRLs as biologically meaningful markers of persistent lesion-edge inflammation with substantial prognostic relevance.

### 5.2 Slowly expanding lesions as markers of chronic lesion expansion

Whereas PRLs emphasize the composition of the lesion edge, SELs capture a different but closely related phenomenon: the gradual outward expansion of established lesions over time. This feature is particularly important because it reflects chronic lesion activity at the structural level. Rather than focusing on acute lesion formation, SEL analysis identifies lesions that continue to enlarge slowly, consistent with persistent damage at the lesion border. In this sense, SELs operationalize one of the core pathological hallmarks of smoldering neuroinflammation—namely, that lesions may remain active long after the acute inflammatory phase has passed (113, 114, 119). Calvi et al. provided one of the key clinical studies in this area by showing that definite SELs account for a substantial proportion of T2 lesions in secondary progressive MS and are associated with neurodegenerative MRI measures as well as clinical worsening (120). This is an important finding because it demonstrates that slow lesion expansion is not a rare or incidental imaging feature, but a common component of progressive disease burden. It also supports the interpretation of SELs as *in vivo* markers of progression-related pathology, linking lesion-level structural dynamics to patient-level disability accumulation. Compared with conventional lesion counts, SELs may therefore offer a more relevant readout of the chronic inflammatory processes that drive long-term tissue injury in progressive MS.

### 5.3 Are PRLs and SELs the same?

The growing literature on PRLs and SELs has naturally raised the question of whether they represent the same lesions viewed through different imaging methods. The most accurate answer at present is that they are related but not identical. Elliott et al. addressed this issue directly at the lesion level and showed that PRLs and SELs overlap only partially (121). Lesions bearing both features tended to exhibit the most severe tissue destruction, whereas many lesions displayed only one of the two characteristics. This argues against a simplistic equivalence model and instead suggests that PRLs and SELs index complementary dimensions of chronic lesion activity. PRLs appear to emphasize lesion-edge composition, especially iron-associated inflammatory rims, whereas SELs more directly reflect the spatial behavior of slow chronic expansion over time. Methodological studies reinforce this distinction. Huang et al. showed that QSM identifies PRLs with greater myelin damage and stronger clinical associations than those detected using phase imaging, underscoring that PRL definition depends heavily on imaging technique and pathological specificity (122). More recently, Gillen et al. further supported the superiority of QSM over phase imaging for identifying chronically active lesions with paramagnetic rims (123). These findings matter because they caution against treating all rim-like lesions as biologically equivalent. They also suggest that PRLs and SELs should not be collapsed into a single imaging category, even if both are informative about chronic activity. A more useful framework is to view them as partially overlapping biomarkers that together improve lesion phenotyping. The relationship between PRLs and SELs should not be reduced to a simple model of partial overlap. PRLs are heterogeneous from a longitudinal imaging perspective, and their biological meaning may depend on lesion age, gadolinium enhancement status, rim persistence, and core–rim evolution (69, 124, 125). Elkady et al. showed that acute gadolinium-enhancing PRLs and chronic non-enhancing PRLs can differ in the longitudinal evolution of rim and core MRI features, indicating that PRLs do not represent a single static lesion state. Similarly, long-term PRL studies indicate that PRLs may appear, persist, or disappear over time, and that these trajectories may have different implications for clinical-trial design and treatment-response monitoring. Therefore, PRLs and SELs should be interpreted as dynamic and partially overlapping lesion-state markers rather than fixed or interchangeable indicators of chronic active lesion activity. From a practical imaging perspective, this partial overlap means that PRLs and SELs should not be ranked simply as more or less sensitive versions of the same biomarker. Instead, PRLs are more closely related to lesion-edge susceptibility and iron-associated inflammatory pathology, whereas SELs capture chronic spatial expansion. Their combined use may therefore improve lesion phenotyping, but discordant PRL-only or SEL-only lesions require careful interpretation.

### 5.4 PET evidence for metabolically active chronic inflammation

An important limitation of MRI-based biomarkers is that they are still indirect readouts of chronic lesion biology. PET imaging adds a complementary dimension by assessing inflammatory metabolism more directly, particularly through translocator protein (TSPO) ligands that reflect activated innate immune cells. In this regard, TSPO-PET has provided some of the strongest *in vivo* evidence that chronic lesion activity in MS is not merely morphological or historical, but metabolically active and clinically meaningful.

Hamzaoui et al. showed using [18F]-DPA-714 PET that an unexpectedly high proportion of MS lesions contain a smoldering inflammatory component and that this component predicts atrophy and clinical deterioration (14). This study was especially important because it shifted the field from viewing chronic lesions as structurally abnormal but possibly inactive, to viewing many of them as metabolically active sites of ongoing inflammation. Polvinen et al. extended this concept by showing that TSPO-detectable chronic active lesions strongly predict future disease progression (126). Taken together, these studies establish that chronic active lesion biology can be identified not only by lesion morphology or susceptibility characteristics, but also by persistent innate immune metabolic activity. This provides particularly strong support for the broader concept of smoldering neuroinflammation and helps validate MRI-based chronic lesion markers against a more biologically proximate imaging measure.

### 5.5 Emerging imaging biomarkers: 3T applicability and broad rim lesions

For chronic lesion biomarkers to influence routine clinical practice, they must be adaptable beyond highly specialized research settings. One important step in this direction has been the demonstration that rim lesions can also be detected at 3T. Vanheule et al. showed that rim lesions identified at 3T retain clinical relevance and may serve as follow-up markers, which is encouraging for translational adoption (127). Although ultra-high-field MRI remains superior for certain applications, the growing feasibility of PRL detection at 3T increases the likelihood that chronic active lesion imaging will become more broadly integrated into clinical workflows and multicenter studies. Another major recent advance is the description of broad rim lesions. Klotz et al. identified broad rim lesions as a new pathological and imaging biomarker associated with rapid disease progression in multiple sclerosis (128). This is a notable development because it suggests that chronic active lesions themselves may not form a uniform class; instead, there may be subtypes with differing biological intensity and prognostic implications. Broad rim lesions may represent a more aggressive end of the chronic active lesion spectrum, potentially capturing lesions with more extensive edge pathology and faster clinical consequences. Their introduction points to the next stage of the field, in which lesion phenotyping may become more granular and more tightly linked to therapeutic stratification.

**Figure 4:**
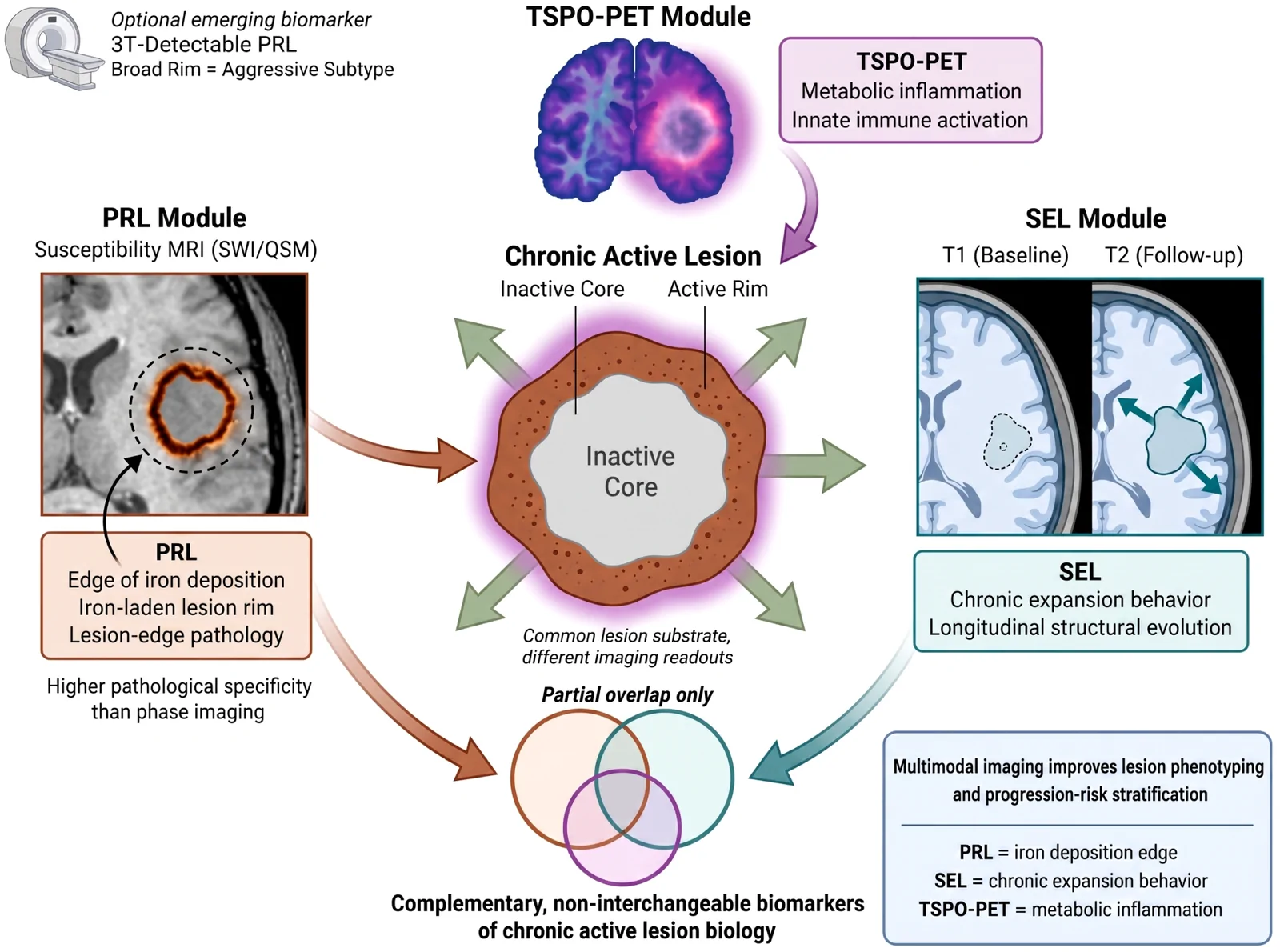
*In vivo* imaging biomarkers of smoldering neuroinflammation in progressive multiple sclerosis. Paramagnetic rim lesions (PRLs), slowly expanding lesions (SELs), and TSPO-PET represent complementary but non-identical imaging biomarkers of chronic active lesion biology. Together, they capture iron-laden lesion rims, slow structural lesion expansion, and persistent innate immune metabolic activity, thereby revealing hidden chronic inflammation that is linked to progression, tissue damage, and worsening clinical outcomes.

Overall, PRLs, SELs, and TSPO-PET together have transformed the study of progressive MS by making chronic active lesion biology increasingly visible *in vivo*. Their greatest value lies not merely in diagnosis, but in reframing disease monitoring around the forms of inflammation that remain active when conventional measures appear quiet. At the same time, the partial overlap among these modalities should be embraced rather than treated as a weakness. It reflects the biological complexity of chronic active lesions and suggests that the most informative approach may be a multimodal one that integrates lesion composition, structural expansion, and inflammatory metabolism. **Figure 4** illustrates PRLs, SELs, and TSPO-PET as complementary *in vivo* biomarkers of chronic active lesions in progressive multiple sclerosis. It highlights a multimodal imaging framework in which lesion-edge composition, structural expansion, and metabolic inflammation are integrated to detect otherwise hidden smoldering neuroinflammation.

### 5.6 Multimodal imaging comparison and practical limitations

Although PRLs, SELs, and TSPO-PET are all used to infer smoldering lesion activity, they differ substantially in what they measure and how readily they can be implemented. PRLs detected by susceptibility-based MRI provide relatively specific information about iron-associated lesion-edge pathology, especially when QSM or ultra-high-field MRI is used. However, PRL detection is sensitive to field strength, sequence parameters, image processing, and reader experience. QSM may improve pathological specificity compared with phase imaging, but it requires optimized acquisition and post-processing pipelines (129–131). By contrast, 3T MRI is more clinically accessible but may have lower sensitivity for subtle rims than 7T MRI. SELs provide a complementary longitudinal measure by identifying established lesions that continue to expand over time. Their strength lies in capturing chronic structural evolution, but they are not specific for iron-laden inflammatory rims and require repeated MRI scans, reliable lesion segmentation, and harmonized algorithms. TSPO-PET offers a more direct readout of innate immune activation and inflammatory metabolism, but it is limited by radiotracer availability, cost, spatial resolution, TSPO genotype effects, and incomplete cellular specificity. Therefore, no single modality should be considered a definitive surrogate for chronic active lesion biology. A multimodal approach combining PRL morphology, SEL dynamics, and PET-defined inflammatory activity may provide a more reliable framework for lesion phenotyping, patient stratification, and treatment-response monitoring.

### 5.7 Technical limitations and biological interpretation of imaging biomarkers

The biological meaning of PRLs, SELs, and TSPO-PET findings depends strongly on the technique used to identify them. PRLs are not direct pathological diagnoses; they are susceptibility-based imaging correlates that are influenced by field strength, spatial resolution, sequence parameters, QSM reconstruction, phase-processing methods, and reader expertise. QSM may improve specificity for iron-associated rims compared with phase imaging, but it remains dependent on post-processing and standardization (132–134). SELs capture longitudinal structural expansion, but they do not directly identify iron-laden inflammatory rims or metabolically active immune cells. Their detection depends on follow-up duration, lesion segmentation, registration accuracy, and algorithmic definitions. TSPO-PET provides a closer readout of inflammatory metabolism, but TSPO signal is not fully cell-type specific and is limited by tracer availability, binding polymorphisms, cost, spatial resolution, and clinical accessibility. Therefore, these modalities should be interpreted as partially overlapping windows onto chronic lesion biology rather than interchangeable measures of the same pathological entity.

## 6 From chronic lesion activity to neuroaxonal loss and disability accumulation

A central question in progressive MS is why chronic active lesions matter clinically. The answer lies in the type of injury they sustain. Smoldering lesions are not simply radiological abnormalities persisting over time; they are sites of ongoing demyelination, axonal stress, oxidative injury, blood–brain barrier disturbance, and failed tissue repair (135–139). Through these processes, they create a chronic lesion microenvironment in which structural damage accumulates gradually but continuously (34). This helps explain why progressive disability can emerge even in the relative absence of acute relapses or new enhancing lesions (140, 141). In effect, chronic active lesions provide a biological bridge between persistent inflammation and long-term neurodegeneration. This relationship is especially important in the context of progression independent of relapse activity (PIRA) (19). If a lesion remains chronically active at its edge, then tissue destruction can continue without the dramatic inflammatory events that typically define overt relapse activity. Over time, the cumulative effect of such lesion-centered injury may extend beyond the lesion itself, influencing white matter tract integrity, cortical connectivity, and broader gray–white matter network function (142). Chronic lesion activity should therefore be viewed not as a localized pathology with limited clinical significance, but as a potential engine of widespread functional decline (34, 141). **Table 4** outlines the clinical consequences of chronic active lesions and the corresponding therapeutic implications, linking lesion-edge pathology to neuroaxonal loss, biomarker changes, disability accumulation, and the limitations of current disease-modifying therapies.

**Table 4:** Clinical consequences and therapeutic implications of chronic active lesions in progressive multiple sclerosis.

Domain	Main consequence	Mechanistic basis	Clinical implication	Representative PMID
Neuroaxonal injury	Progressive axonal stress and degeneration	Persistent demyelination, oxidative stress, mitochondrial dysfunction, lesion-edge inflammation	Chronic active lesions act as a biological bridge between inflammation and neurodegeneration	37276054
Structural tissue damage	Ongoing white matter and network-level injury	Slow lesion expansion, failed repair, chronic border activity	Lesion burden may influence broader gray–white matter disconnection and long-term decline	35277438
Biomarker change	Increased serum neurofilament light chain (sNfL)	Ongoing axonal injury caused by chronic lesion activity	Supports the biological relevance of PRL/SEL burden beyond structural imaging alone	34088875
Disability accumulation	Gradual worsening despite relapse control	Persistent CNS-compartmentalized inflammation and non-resolving lesions	Helps explain progression independent of relapse activity (PIRA)	38366920
Prognostic value	Higher PRL/SEL/chronic lesion burden predicts worse outcome	More extensive chronic active pathology reflects higher progression-prone disease state	Chronic lesion biomarkers may improve risk stratification	40357663
Therapeutic limitation of current DMTs	Incomplete control of progression despite relapse suppression	Current DMTs are more effective against peripheral acute inflammation than CNS-trapped chronic lesion activity	Explains why reducing relapses does not fully prevent disability accumulation	37437310
Partial lesion modulation	Some therapies may attenuate chronic lesion activity, but incompletely	Limited impact on lesion-edge inflammatory networks	Suggests that current treatments only partly modify smoldering pathology	35046083
Future therapeutic direction	Need lesion-directed and compartment-targeted therapy	Target resident innate immune cells, iron-related stress, glial crosstalk, and repair failure	Supports a shift from relapse suppression toward progression prevention	37437310
Role in clinical trials	Potential enrichment and endpoint biomarker	PRL, SEL, and TSPO-PET identify ongoing progression-driving biology	Multimodal chronic lesion imaging may improve trial design and treatment monitoring	37349108

### 6.1 Chronic demyelination and axonal degeneration

The most direct route by which smoldering lesions contribute to progression is through the continued coupling of chronic demyelination and axonal degeneration (143–146). Lesion-edge inflammation sustains a state in which myelin breakdown remains incomplete and axons remain exposed to persistent metabolic and inflammatory stress. In such lesions, the border is a zone of active tissue compromise rather than simple scar formation. This is important because chronically demyelinated axons are particularly vulnerable to conduction failure, mitochondrial stress, and eventual degeneration. The longer this non-resolving lesion state persists, the greater the likelihood of irreversible neurological loss.

Imaging work supports this interpretation. Choi et al. used multiparametric 7T MRI to characterize blood–brain barrier integrity, demyelination, and neurodegenerative changes in PRLs. Although overt blood–brain barrier breakdown was not detected, PRLs showed imaging features consistent with greater demyelination, cell loss, and possible disruption of tissue anisotropy, and their presence was associated with subsequent disability progression (78). This indicates that PRLs capture lesion states in which several forms of damaging biology converge rather than a single isolated mechanism (120). Likewise, the association of SELs with neurodegenerative MRI markers in secondary progressive MS, as shown by Calvi et al., suggests that slowly expanding lesions are embedded within broader tissue degeneration rather than acting as benign imaging curiosities. Together, these observations strengthen the view that chronic lesion activity causes harm because it sustains the conditions for ongoing structural injury.

### 6.2 Biomarker links: sNfL and imaging burden

A particularly compelling feature of the current literature is the emerging link between chronic lesion imaging and fluid biomarkers of axonal damage. Maggi et al. reported that chronic white matter inflammation, reflected by PRL-related imaging findings, is associated with increased serum neurofilament light chain (sNfL) levels and with greater disease severity in non-acute MS (26). This study is important because it connects lesion-level chronic activity to a systemic indicator of neuroaxonal injury, thereby moving the field beyond descriptive imaging correlations. It suggests that chronic active lesions are biologically consequential enough to leave a measurable signature in circulating biomarkers of neural damage. This bridge between imaging and fluid biomarkers also has broader implications. If PRL or SEL burden correlates with markers such as sNfL, then chronic lesion activity may be trackable not only structurally but also biologically, offering a more integrated way to monitor progression-prone disease (147–150). Such a framework would be particularly valuable in progressive MS, where standard MRI measures often underestimate clinically relevant ongoing damage. Although further work is needed to refine these associations longitudinally, the existing evidence supports the interpretation of chronic lesion biomarkers as more than structural surrogates: they are linked to active neuroaxonal injury. However, the temporal sequence between PRL emergence and sNfL elevation remains incompletely resolved. Current evidence supports a biological link between chronic white matter inflammation and axonal injury, but does not yet establish a uniform sequence in which PRL formation consistently precedes measurable sNfL increase in all patients. Longitudinal studies combining serial susceptibility imaging and repeated fluid biomarker measurements are therefore needed to define whether sNfL acts as a downstream marker of chronic lesion activity or a broader indicator of concurrent neuroaxonal injury.

### 6.3 Lesion burden as a predictor of worsening MS

At the patient level, one of the strongest arguments for the importance of chronic active lesions is their association with future disability accumulation. Reeves et al. reported that PRLs predict greater long-term relapse rates and more severe long-term clinical progression, consistent with the idea that they identify a more aggressive disease course (151). Bagnato et al. further showed that chronic active lesion burden is frequently associated with disease progression and disability accumulation in worsening MS. These findings suggest that chronic lesion burden is not merely a marker of past disease severity, but an indicator of ongoing progression risk. The same logic applies to SELs. Calvi et al. demonstrated that SEL burden is related to clinical worsening in secondary progressive MS, reinforcing the view that structural evidence of slow lesion expansion carries prognostic information. When considered together, PRL burden, SEL burden, and PET-detectable chronic activity converge on the same general conclusion: the more evidence there is for non-resolving lesion pathology, the more likely the patient is to experience continuing clinical decline (152, 153). This is one of the most important messages of the field, because it reframes chronic active lesions as a clinically actionable component of disease assessment rather than a specialized research observation. However, these associations should be interpreted as longitudinal prognostic links rather than definitive proof that imaging-detected chronic active lesions always temporally precede and cause subsequent EDSS worsening.

**Figure 5:**
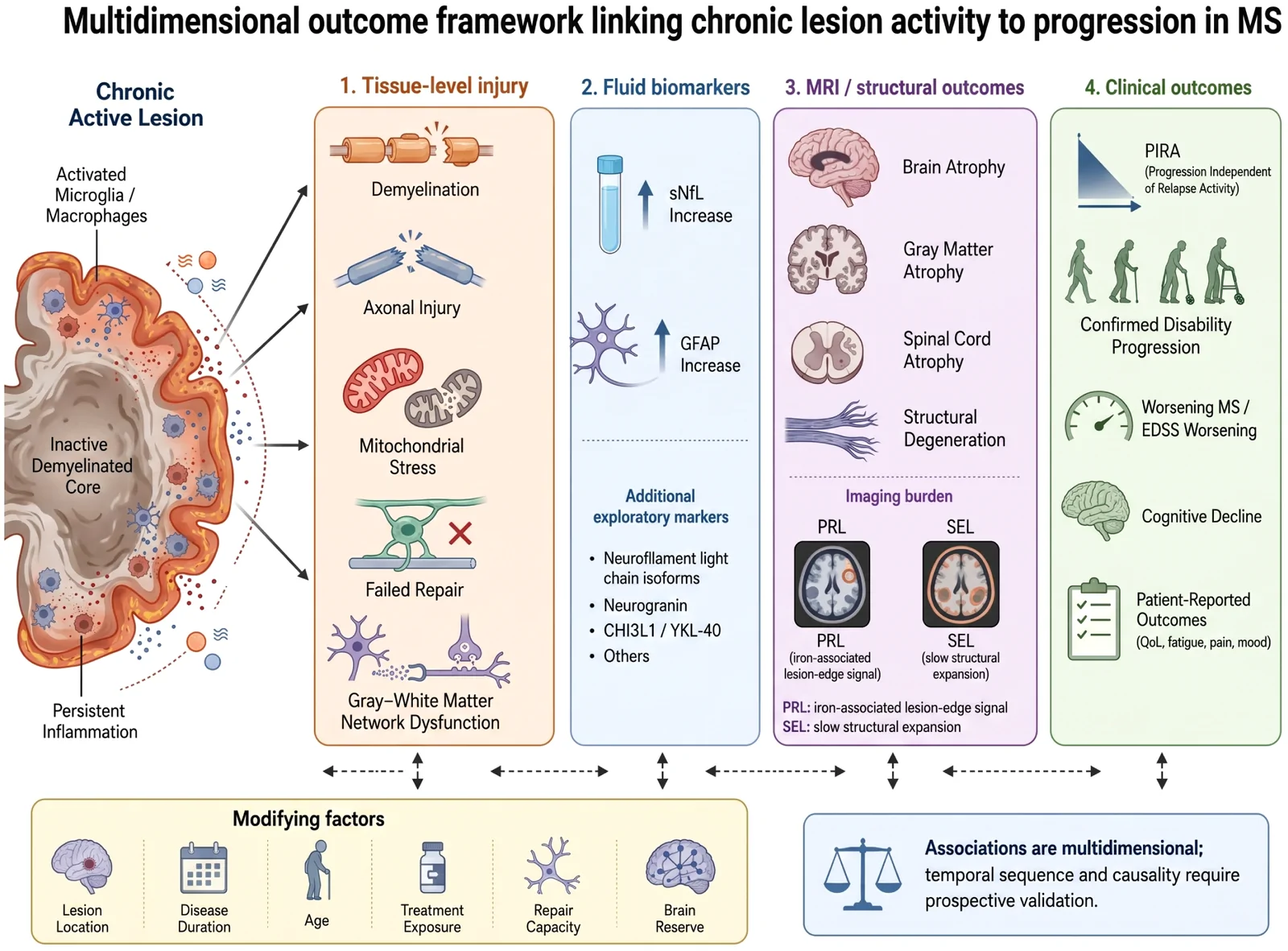
Multidimensional outcome framework for chronic lesion activity and disease progression. Chronic lesion activity may be associated with neuroaxonal injury, fluid biomarker changes, MRI-defined neurodegeneration, and clinical worsening, but these relationships should not be interpreted as a fixed linear sequence. sNfL reflects one dimension of ongoing neuroaxonal injury, whereas brain atrophy, gray matter atrophy, spinal cord atrophy, confirmed disability progression, cognitive decline, and patient-reported outcomes provide complementary measures of progression. Lesion location, disease duration, age, treatment exposure, repair capacity, and baseline brain reserve may modify how chronic lesion activity translates into disability.

In summary, chronic active lesions help explain how inflammation can continue to drive disability even when classical acute disease activity appears suppressed. By sustaining demyelination, axonal injury, blood–brain barrier abnormalities, iron-related tissue stress, and failed repair, these lesions generate a form of chronic inflammatory damage that is especially relevant to long-term progression (154–156). Their growing association with sNfL elevation, structural degeneration, and clinical worsening makes them central to any modern account of how progressive MS accumulates disability over time. **Figure 5** illustrates how chronic active lesions act as a biological bridge between persistent inflammation and long-term neurodegeneration in progressive multiple sclerosis. It highlights lesion-edge-driven demyelination, axonal injury, and failed repair as key processes linking chronic lesion burden to sNfL elevation, disability accumulation, and progression independent of relapse activity. However, chronic active lesion burden does not fully capture the tissue basis of disability accumulation. Progressive worsening may also reflect diffuse injury in normal-appearing white matter, cortical and deep gray matter damage, leukocortical lesion burden, brain and spinal cord atrophy, synaptic loss, and network disconnection. These abnormalities may occur alongside focal PRLs or SELs, but they may also progress when focal inflammatory activity is limited. Therefore, PRL and SEL burden should be viewed as markers of a progression-prone focal lesion phenotype rather than comprehensive biomarkers of all progressive tissue injury.

### 6.4 Beyond sNfL: atrophy, clinical endpoints, and patient-reported outcomes

While sNfL is useful for capturing neuroaxonal injury, the translational relevance of chronic active lesion biology should also be evaluated using established imaging and clinical outcome measures. Brain atrophy, particularly gray matter atrophy, is one of the most clinically meaningful MRI markers of neurodegeneration in progressive MS. Unlike sNfL, which reflects ongoing axonal injury at a given time point, brain and gray matter atrophy integrate cumulative tissue loss and may better capture long-term neurodegenerative burden. Therefore, the relationship between PRL or SEL burden and atrophy measures is essential for determining whether chronic lesion activity contributes to clinically relevant tissue loss. Clinical endpoints are equally important. Confirmed disability progression, EDSS worsening, timed walking measures, upper-limb function, cognitive decline, and patient-reported outcomes provide complementary information that cannot be inferred from sNfL alone (157, 158). These endpoints are necessary to validate whether chronic active lesion biomarkers have practical value for prognosis, patient stratification, and treatment-response monitoring. Future studies should therefore combine PRL, SEL, and TSPO-PET assessment with longitudinal atrophy measures, repeated fluid biomarkers, and standardized clinical and patient-reported outcomes.

### 6.5 Temporal sequence and predictive value of chronic lesion biomarkers

The predictive value of PRLs and SELs depends on whether these imaging abnormalities precede subsequent biological and clinical worsening. Longitudinal studies provide partial support for such a temporal framework. PRLs can persist for years after lesion formation and have been associated with later clinical and radiological progression, suggesting that rim-positive lesions may mark a chronic lesion state with future prognostic relevance. Similarly, longitudinal SEL analyses indicate that slow lesion expansion is associated with neurodegenerative MRI changes and disability worsening in secondary progressive MS, supporting the view that SELs capture an ongoing process rather than a static lesion feature (159, 160). PET studies further suggest that metabolically active chronic inflammatory lesions may predict future disease progression. Nevertheless, the exact sequence linking PRL or SEL appearance to sNfL elevation, accelerated brain atrophy, and EDSS worsening remains insufficiently defined. Available studies more strongly support prognostic association than direct temporal causality. Therefore, PRLs, SELs, and TSPO-PET activity should currently be regarded as promising predictive biomarkers, but their use as validated surrogate endpoints requires longitudinal cohorts with harmonized imaging protocols, repeated sNfL/GFAP measurements, brain-volume assessment, and standardized disability outcomes.

## 7 Why current disease-modifying therapies incompletely control smoldering neuroinflammation

Compared with pathological and imaging evidence, treatment-response evidence for chronic active lesion modification remains more limited and should be interpreted cautiously. The therapeutic revolution in multiple sclerosis (MS) has been built largely on the successful suppression of acute inflammatory activity. Many current disease-modifying therapies (DMTs) substantially reduce relapse frequency, diminish new gadolinium-enhancing lesions, and limit overt focal inflammatory activity on conventional MRI (161–163). Yet even under these conditions, many patients—especially those with progressive disease—continue to accumulate disability. This discrepancy has become one of the most important unresolved questions in modern MS therapeutics. It suggests that while current DMTs are effective against the inflammatory biology of relapse formation, they are much less effective against the chronic lesion-centered and compartmentalized inflammatory processes that sustain progression. In other words, relapse control and progression control are related but not equivalent therapeutic achievements.

The growing literature on chronic active lesions provides a compelling explanation for this therapeutic gap. Progressive MS is increasingly characterized by inflammation that is retained within the central nervous system (CNS), spatially organized at lesion edges and other protected niches, and sustained by resident innate immune cells rather than dominated by recurrent waves of peripheral adaptive immune infiltration (164–166). Under such conditions, therapies that primarily suppress peripheral immune activation may leave substantial components of lesion-edge pathology relatively intact. Imaging studies using PRLs, TSPO-PET, and related biomarkers reinforce this concern by showing that chronic lesion activity often persists despite otherwise effective anti-inflammatory treatment. This implies that current DMTs may reduce the generation of new lesions without fully extinguishing the biology of pre-existing chronic active lesions that continue to drive tissue injury over time. However, this conclusion should not be interpreted as a uniform failure of all DMTs. Available evidence is still limited and appears to be biomarker- and drug-class-specific, with different data emerging for anti-CD20 therapy, S1P receptor modulators, and dimethyl fumarate.

### 7.1 Peripheral immune suppression does not fully reach compartmentalized CNS inflammation

One of the clearest demonstrations of this limitation comes from B-cell depletion therapy. Anti-CD20 treatments are among the most effective therapies for suppressing acute inflammatory activity in MS, yet their effects on chronic active lesion biology appear incomplete. Maggi et al. showed that B-cell depletion does not resolve chronic active lesions, despite its robust capacity to suppress relapsing inflammatory activity (167). This finding is particularly relevant to anti-CD20 therapy, because it suggests that strong suppression of B-cell-driven acute inflammatory activity does not necessarily eliminate established PRLs or chronic active lesion biology. Therefore, the persistence of PRLs under B-cell depletion should be interpreted as evidence of incomplete control of pre-existing CNS-compartmentalized lesion activity, rather than as evidence that anti-CD20 therapy lacks anti-inflammatory efficacy (168–170). The distinction is important because anti-CD20 therapies may prevent new inflammatory activity while having a more limited effect on established lesion-edge inflammation. This is a particularly important observation because it reveals a therapeutic dissociation between controlling overt focal inflammation and eliminating progression-associated chronic lesion pathology. If chronic active lesions persist under highly effective peripheral immune suppression, then their maintenance must depend, at least in part, on inflammatory circuits that are not fully accessible to current DMT strategies. This therapeutic uncoupling is highly consistent with the broader concept of compartmentalized CNS inflammation. Once chronic active lesions are established, the relevant cellular networks may become partly self-sustaining within the CNS through interactions among resident microglia, infiltrating but locally retained lymphocytes, astrocytes, and iron-associated inflammatory pathways. Under such conditions, even profound peripheral immune suppression may have only limited impact on the lesion-edge microenvironment. This helps explain why progression can continue despite apparent success by conventional therapeutic benchmarks. It also suggests that the most clinically meaningful inflammatory processes in progressive MS may not be those that are easiest to suppress pharmacologically, but rather those that remain active within protected CNS compartments (171–173). Further support for this interpretation comes from PET-based imaging studies. Hamzaoui et al. and Polvinen et al. showed that chronic inflammatory lesion activity, detectable by TSPO-PET, is associated with brain atrophy, disability worsening, and future disease progression. These observations imply that the biologically active lesions most relevant to progression are not adequately captured by conventional inflammatory monitoring alone and may persist despite therapies that effectively reduce new lesion formation. Together, these data argue that progression in MS cannot be fully controlled without addressing CNS-trapped inflammatory processes that remain operational after acute inflammation has been pharmacologically restrained.

### 7.2 Partial lesion modulation is possible but incomplete

Although the limitations of current DMTs are increasingly apparent, existing evidence does suggest that some degree of chronic lesion modulation is possible. Zinger et al. reported that dimethyl fumarate (DMF) was associated with reduced susceptibility changes in chronic active lesions, consistent with a decrease in inflammatory activity at the lesion edge (174). DMF therefore represents an example of partial biomarker modulation rather than complete lesion resolution. By contrast, evidence for S1P receptor modulators is more often related to lesion formation, brain-volume loss, or longitudinal lesion evolution, and less directly to established PRL resolution. Thus, available data suggest that different DMT classes may influence different aspects of smoldering pathology: anti-CD20 therapy may incompletely resolve established PRLs, DMF may attenuate QSM-detectable rim susceptibility, and S1P modulators may be more relevant to broader longitudinal inflammatory or degenerative activity (175, 176). Direct evidence linking these treatments to TSPO-PET signal reduction remains limited.

However, the key point is that such modulation appears incomplete. The current evidence does not support the conclusion that existing DMTs reliably extinguish the cellular and molecular programs sustaining chronic active lesions. Instead, the available data suggest a more modest scenario in which some therapies may reduce aspects of lesion activity without fundamentally dismantling the compartmentalized inflammatory architecture that underlies progression. This distinction is crucial. It means that therapeutic benefit in progressive MS may reflect degrees of partial biological attenuation rather than true lesion resolution, and it underscores the need for more precise treatment goals in the progressive phase of disease. In this light, the persistence of chronic active lesions should not be interpreted as evidence that therapy has completely failed, but rather as evidence that the therapeutic target has changed. Acute inflammatory lesion formation and chronic lesion persistence are not identical processes, and their differential responsiveness to treatment should not be surprising. The challenge for the next phase of therapeutic development is therefore not simply to intensify relapse suppression, but to design interventions that are biologically matched to the lesion-edge and compartmentalized inflammatory programs that current therapies only partly influence.

### 7.3 Implications for next-generation treatment design

These findings have important implications for how future treatments for progressive MS should be conceptualized. First, therapeutic strategies must move beyond an almost exclusive emphasis on peripheral adaptive immunity and engage more directly with resident innate immune activation within the CNS. Chronic active lesions are sustained by lesion-edge microglia and macrophage-lineage cells, astrocyte–immune cross-talk, iron-related inflammatory stress, and incomplete tissue resolution (177–180). These processes are unlikely to be adequately controlled by therapies designed primarily to prevent immune-cell entry into the CNS or to deplete circulating lymphocyte populations. Second, future treatment design should increasingly incorporate chronic lesion biomarkers as indicators of therapeutic relevance. If PRLs, SELs, and TSPO-PET-detectable lesions identify disease-driving pathology that remains active under standard treatment, then they may be more informative than conventional MRI endpoints for evaluating therapeutic success in progressive MS. A therapy that reduces new lesion counts but leaves chronic lesion burden unchanged may be less meaningful for long-term progression than one that directly attenuates lesion-edge activity. This argues for a substantial shift in both trial design and therapeutic thinking—from a paradigm centered on relapse suppression to one focused on the biological control of persistent CNS-trapped inflammation.

## 8 Therapeutic landscape and emerging strategies for smoldering inflammation

Therapeutic strategies for smoldering inflammation should be discussed according to their current level of evidence. Approved disease-modifying therapies (DMTs) remain important for suppressing relapses, new focal inflammatory lesions, and early inflammatory activity, but their ability to resolve established chronic active lesions remains limited or uncertain. Emerging approaches, including BTK inhibitors, advanced B-cell-directed strategies, CAR-T cell therapy, and imaging-guided trial designs, may be more directly relevant to progressive biology, but most still require prospective validation using PRL, SEL, TSPO-PET, atrophy, and disability endpoints (181–183). If chronic active lesions are a central pathological substrate of progression in MS, then future therapy must be designed around the biology of these lesions rather than around relapse suppression alone. The emerging literature suggests that several therapeutic directions are especially promising. These include targeting resident innate immune activation, interfering with iron-related lesion biology, modulating glial cross-talk, addressing meningeal and compartmentalized inflammation, promoting remyelination and tissue repair, and incorporating chronic lesion biomarkers into patient stratification and trial design (184–187). Collectively, these approaches signal a transition toward a more mechanism-based therapeutic framework for progressive MS. A key principle underlying this shift is that progression should not be treated as a passive aftermath of earlier inflammatory damage. Rather, it should be understood as an active pathological state in which chronic lesion-edge inflammation, metabolic stress, and failed repair remain biologically engaged. This distinction is crucial because it broadens the therapeutic horizon: instead of asking only how to reduce new acute lesions, the field must now ask how to interrupt the persistence of existing lesion pathology and how to prevent chronic inflammatory lesions from continuing to expand, destabilize tissue, and drive neuroaxonal loss. Imaging biomarkers such as PRLs, SELs, TSPO-PET activity, and newer lesion phenotypes like broad rim lesions may be particularly useful in this effort, both as biological readouts and as tools for precision intervention.

### 8.1 Approved DMTs and early prevention of chronic lesion establishment

Although currently approved DMTs may incompletely control established chronic active lesions, their role should not be dismissed. Early and effective suppression of acute inflammatory activity may reduce the formation of new lesions and thereby decrease the pool of lesions that could later evolve into chronic active or rim-positive lesions. This preventive effect is conceptually different from resolving already established PRLs or SELs (132, 188, 189). Therefore, DMTs may be most relevant at two levels: preventing new inflammatory lesion formation early in the disease course and limiting additional lesion substrate, while having more limited effects on entrenched lesion-edge inflammation once chronic active lesions have developed. However, direct evidence that early DMT exposure specifically prevents future PRL formation or chronic active lesion establishment remains limited. Future studies should test whether early high-efficacy treatment reduces the incidence, persistence, or expansion of PRLs and SELs using standardized longitudinal susceptibility MRI and lesion-expansion analyses.

### 8.2 BTK inhibitors: targeting B-cell and myeloid components of smoldering inflammation

BTK inhibitors are among the most relevant near-term therapeutic candidates for smoldering inflammation because BTK signaling is involved in B-cell activation and innate myeloid-cell function. This dual biological rationale is particularly relevant to chronic active lesion biology, where compartmentalized B-cell responses, microglial activation, and lesion-edge myeloid inflammation may coexist. Unlike broader relapse-focused immunosuppression, brain-penetrant BTK inhibition may theoretically modulate both peripheral immune activity and CNS-compartmentalized inflammatory processes (62, 190, 191). Recent clinical data have strengthened interest in this approach, particularly for progressive MS. In the phase 3 HERCULES trial, tolebrutinib was evaluated in non-relapsing secondary progressive MS and was reported to reduce the risk of disability progression compared with placebo. However, this should not be interpreted as definitive proof that BTK inhibition resolves PRLs or chronic active lesions, because lesion-level imaging endpoints require dedicated validation. Other BTK inhibitors, including fenebrutinib, are also being evaluated in progressive MS; the FENtrepid phase 3 trial compares fenebrutinib with ocrelizumab in primary progressive MS. Therefore, BTK inhibitors should be presented as promising but still incompletely validated agents for smoldering lesion biology.

### 8.3 Cellular and B-cell-directed immune-reset strategies

More intensive immune-reset strategies, including CAR-T cell therapy, are emerging as experimental approaches for treatment-refractory progressive MS. Their rationale is based on deeper depletion or reprogramming of pathogenic B-cell compartments than conventional anti-CD20 therapy. Early-phase trials are evaluating anti-CD19 CAR-T approaches such as KYV-101 in non-relapsing or progressive forms of MS and in treatment-refractory progressive MS (192, 193). These studies are primarily designed to assess feasibility and safety, including cytokine release syndrome, neurotoxicity, and immune reconstitution, rather than to establish efficacy against chronic active lesions. At present, CAR-T therapy should therefore be mentioned as an emerging experimental strategy, not as a near-term standard treatment. Its relevance to smoldering inflammation will depend on whether future trials demonstrate effects on compartmentalized CNS inflammation, PRL or SEL evolution, TSPO-PET activity, neurodegeneration, and confirmed disability progression.

### 8.4 Targeting resident innate immunity

One of the most compelling future directions is the direct targeting of resident innate immune cells within chronic active lesions. Multiple lines of evidence now indicate that lesion-edge microglia and macrophage-lineage cells are central effectors of smoldering injury (194–197). These cells integrate phagocytosis, inflammatory signaling, oxidative stress, and iron handling, thereby sustaining the chronic lesion microenvironment. Targeting these populations, or the signaling programs that maintain their pathogenic activation states, may therefore offer a more rational approach to progressive MS than simply intensifying peripheral immune suppression. At the same time, any strategy aimed at resident innate immunity will need to account for functional heterogeneity. Microglia and myeloid cells can contribute both to injury and to repair, and broad suppression of these populations may carry unintended consequences. This suggests that future therapies should aim not merely to inhibit innate immune cells globally, but to reprogram lesion-edge myeloid states away from chronic inflammatory persistence and toward resolution-promoting functions. Such approaches may be particularly attractive when combined with imaging biomarkers that identify patients with a high burden of chronic active lesions most likely to benefit from lesion-directed intervention. At present, this strategy remains largely mechanistic and requires clinical imaging evidence showing that modulation of resident innate immune states can reduce PRL persistence, SEL expansion, or PET-defined inflammatory activity.

### 8.5 Targeting iron-related lesion biology

Iron-related lesion biology represents another promising therapeutic axis. Hofmann et al. showed that iron accumulation at chronic active lesion edges is linked to specific myeloid cell programs, indicating that iron handling is embedded within the biology of lesion persistence rather than simply reflecting prior tissue damage. This raises the possibility that iron metabolism, redox stress, and associated inflammatory pathways may be actionable features of progression-driving lesions. Therapies capable of reducing iron-related oxidative injury or altering the pathogenic iron-handling phenotype of lesion-edge myeloid cells could, in principle, attenuate the self-sustaining inflammatory stress of PRLs and related lesions (198). The recent description of broad rim lesions adds urgency to this line of thought. Klotz et al. identified broad rim lesions as a pathological and imaging biomarker associated with more rapid progression, suggesting that not all chronic active lesions are biologically equivalent. If broad rim lesions indeed represent a more aggressive inflammatory-metabolic phenotype, then lesion subclassification may help identify patients in whom iron-related or lesion-edge-targeted therapies are especially relevant. This kind of stratified lesion biology could become an important component of precision therapeutic development in progressive MS. Although iron-related lesion biology is strongly linked to PRL formation, therapeutic targeting of iron-handling pathways remains speculative until prospective studies demonstrate measurable effects on rim evolution, tissue injury, or clinical progression.

### 8.6 Imaging-guided patient stratification and trial enrichment

A major obstacle in progressive MS trials has been the biological heterogeneity of enrolled populations. Not all patients with progressive disease are driven to the same extent by chronic active lesions, and failure to distinguish among progression mechanisms may dilute therapeutic signals. In this setting, chronic lesion imaging biomarkers offer a major opportunity. PRLs, SELs, and TSPO-PET-detectable lesions can help identify patients in whom progression is more strongly linked to smoldering inflammatory pathology (199–201). This could improve trial enrichment, increase mechanistic precision, and allow therapies to be tested in the populations most likely to respond. These biomarkers may also serve as pharmacodynamic endpoints. Reeves et al. showed that PRL evolution is linked to clinical and radiologic progression, suggesting that changes in PRL status may provide a meaningful readout of therapeutic effect. Likewise, TSPO-PET studies by Hamzaoui et al. and Polvinen et al. suggest that imaging of chronic innate immune activation may be used to monitor whether an intervention truly modifies the biology of chronic active lesions. Moving forward, one of the most important shifts in progressive MS trial design may be the adoption of chronic lesion biomarkers not merely as descriptive measures, but as integral tools for both patient selection and endpoint definition. Compared with lesion-directed therapeutic targeting, this application is closer to clinical translation because PRLs, SELs, and TSPO-PET can already be measured in longitudinal imaging cohorts, although standardization is still required.

### 8.7 Moving from relapse suppression to progression prevention

Ultimately, the broader therapeutic challenge in MS is conceptual as much as technical. The field has been extraordinarily successful in developing therapies that suppress acute inflammatory activity, but progression remains insufficiently controlled because its biology is only partly addressed by this strategy. A more effective framework for the future will require shifting therapeutic goals from relapse suppression alone to progression prevention through direct engagement with chronic lesion biology. This means addressing not only inflammation, but also lesion-edge persistence, glial cross-talk, iron-driven oxidative stress, compartmentalized meningeal and cortical inflammation, and failed repair (173, 202). Such a shift also requires greater integration between mechanistic pathology, imaging, and clinical therapeutics. The most informative future strategies are likely to be those that combine lesion-directed biological targets with biomarkers capable of identifying the relevant disease state *in vivo*. In this sense, PRLs, SELs, TSPO-PET activity, and related lesion phenotypes are not merely descriptive tools; they are part of the therapeutic future of progressive MS. They provide the means to redefine disease activity, stratify patients more intelligently, and evaluate whether therapies are truly modifying the pathological processes that underlie long-term neurological decline.

### 8.8 Future research roadmap

Future research should move from descriptive associations toward testable frameworks. First, imaging protocols for PRLs, SELs, and TSPO-PET should be standardized across field strengths, acquisition sequences, QSM reconstruction methods, lesion segmentation pipelines, PET tracers, and reporting criteria (110, 203). Second, longitudinal cohorts should combine serial MRI/PET, repeated fluid biomarkers such as sNfL and GFAP, brain and spinal cord atrophy measures, and standardized disability outcomes to clarify temporal relationships. Third, imaging-pathology validation is needed to determine how PRLs, SELs, and PET-positive lesions correspond to pathologically defined chronic active lesions. Fourth, clinical trials should evaluate whether chronic lesion biomarkers can serve as enrichment tools, pharmacodynamic readouts, or exploratory endpoints without prematurely treating them as validated surrogate outcomes. This roadmap would help shift the field from narrative description to critical, testable, and clinically actionable evidence.

## 9 Conclusions

Smoldering neuroinflammation is increasingly recognized as a central substrate of progression in multiple sclerosis. Rather than representing the passive aftermath of earlier inflammatory injury, chronic active lesions embody a persistent, biologically active state in which lesion-edge inflammation, compartmentalized CNS immune activity, iron-related stress, and failed repair continue to drive tissue damage over time. This framework helps explain why disability may accumulate even when relapses are controlled and conventional MRI appears relatively stable. Chronic active lesions are not only biologically consequential, but also increasingly measurable *in vivo*. PRLs, SELs, and TSPO-PET-defined chronic inflammatory lesions have transformed the study of progressive MS by making previously hidden pathology visible. Although these biomarkers overlap only partially, together they reveal a disease state characterized by spatially compartmentalized inflammation, chronic lesion persistence, and ongoing neuroaxonal injury. Their growing associations with atrophy, serum neurofilament light chain elevation, and long-term disability progression underscore their importance as both mechanistic and clinical indicators of progression-driving pathology. Future therapies must therefore move beyond relapse control and directly target lesion-edge biology and CNS-trapped inflammation. A next-generation therapeutic framework for progressive MS will likely require a combination of approaches: reprogramming resident innate immune cells, targeting iron-related inflammatory stress, addressing meningeal and compartmentalized inflammation, promoting repair, and integrating chronic lesion biomarkers into patient stratification and trial design. In this way, the study of smoldering neuroinflammation does more than refine our understanding of progressive MS—it redefines the therapeutic agenda for preventing disability accumulation in the disease.

## References

[1] Albelo-Martínez M Rizvi S . Progressive multiple sclerosis: evaluating current therapies and exploring future treatment strategies. Neurotherapeutics. (2025) 22:e00601. doi: 10.1016/j.neurot.2025.e00601 40345951 PMC12418453

[2] Carlson AK Fox RJ . Pathophysiology, diagnosis, treatment and emerging neurotherapeutic targets for progressive multiple sclerosis: the age of PIRA. Neurol Clin. (2024) 42:39–54. doi: 10.1016/j.ncl.2023.07.002 37980122

[3] Picone P Nuzzo D . Promising treatment for multiple sclerosis: mitochondrial transplantation. Int J Mol Sci. (2022) 23. doi: 10.3390/ijms23042245 35216361 PMC8877878

[4] Hochstetler A Lehtinen MK . Choroid plexus as a mediator of CNS inflammation in multiple sclerosis. Mult Scler. (2024) 30:19–23. doi: 10.1177/13524585241292974 39503321 PMC11634642

[5] Ransohoff RM . Multiple sclerosis: role of meningeal lymphoid aggregates in progression independent of relapse activity. Trends Immunol. (2023) 44:266–75. doi: 10.1016/j.it.2023.02.002 36868982

[6] Williams T Zetterberg H Chataway J . Neurofilaments in progressive multiple sclerosis: a systematic review. J Neurol. (2021) 268:3212–22. doi: 10.1007/s00415-020-09917-x 32447549 PMC8357650

[7] Gruchot J Reiche L Chan A Hoepner R Küry P . Human endogenous retrovirus type-W and multiple sclerosis-related smoldering neuroinflammation. Neural Regener Res. (2025) 20:813–4. doi: 10.4103/nrr.nrr-d-24-00121 38886951 PMC11433918

[8] Kukanja P Langseth CM Rubio Rodríguez-Kirby LA Agirre E Zheng C Raman A . Cellular architecture of evolving neuroinflammatory lesions and multiple sclerosis pathology. Cell. (2024) 187:1990–2009.e1919. doi: 10.1016/j.cell.2024.02.030 38513664

[9] Niedziela N Nowak-Kiczmer M Malciene L Stasiołek M Niedziela JT Czuba ZP . Serum vitamin D3 as a potential biomarker for neuronal damage in smoldering multiple sclerosis. Int J Mol Sci. (2024) 25. doi: 10.3390/ijms251910502 39408830 PMC11476431

[10] Absinta M Maric D Gharagozloo M Garton T Smith MD Jin J . A lymphocyte-microglia-astrocyte axis in chronic active multiple sclerosis. Nature. (2021) 597:709–14. doi: 10.1038/s41586-021-03892-7 34497421 PMC8719282

[11] Furlan R Schaedelin S Frederiksen JL Watanabe M Isobe N Piehl F . Granulocyte and astrocyte markers distinguish MOG-antibody disease and neuromyelitis optica from multiple sclerosis. Brain. (2026) 149:1319–31. doi: 10.1093/brain/awaf345 40988129 PMC13058455

[12] Kadowaki A Wheeler MA Li Z Andersen BM Lee HG Illouz T . CLEC16A in astrocytes promotes mitophagy and limits pathology in a multiple sclerosis mouse model. Nat Neurosci. (2025) 28:470–86. doi: 10.1038/s41593-025-01875-9 40033124 PMC12039076

[13] Giordano A Stridh P Preziosa P Pisa M Lerma-Martin C Sorosina M . A HIF1A variant impacts long-term disability and smoldering inflammation in multiple sclerosis. Acta Neuropathol. (2026) 151:12. doi: 10.1007/s00401-026-02984-w 41639279

[14] Hamzaoui M Garcia J Boffa G Lazzarotto A Absinta M Ricigliano VAG . Positron emission tomography with [(18) F]-DPA-714 unveils a smoldering component in most multiple sclerosis lesions which drives disease progression. Ann Neurol. (2023) 94:366–83. doi: 10.1002/ana.26657 37039158

[15] Muñoz González G BA TH Bugiani M Plemel JR Schenk GJ Kooij G . A focus on the normal-appearing white and gray matter within the multiple sclerosis brain: a link to smoldering progression. Acta Neuropathol. (2025) 150:16. doi: 10.1007/s00401-025-02923-1 40783892 PMC12336099

[16] Lassmann H . Pathogenic mechanisms associated with different clinical courses of multiple sclerosis. Front Immunol. (2018) 9:3116. doi: 10.3389/fimmu.2018.03116 30687321 PMC6335289

[17] Iwanowski P Losy J . Immunological differences between classical phenothypes of multiple sclerosis. J Neurol Sci. (2015) 349:10–4. doi: 10.1016/j.jns.2014.12.035 25586536

[18] Baba C Abasiyanik Z Simsek Y Ozdogar AT Sagici O Ozakbas S . Predictors of relapse severity in multiple sclerosis. Acta Neurol Belg. (2024) 124:581–9. doi: 10.1007/s13760-023-02456-y 38238606

[19] Kappos L Wolinsky JS Giovannoni G Arnold DL Wang Q Bernasconi C . Contribution of relapse-independent progression vs relapse-associated worsening to overall confirmed disability accumulation in typical relapsing multiple sclerosis in a pooled analysis of 2 randomized clinical trials. JAMA Neurol. (2020) 77:1132–40. doi: 10.1001/jamaneurol.2020.1568 32511687 PMC7281382

[20] Müller J Cagol A Lorscheider J Tsagkas C Benkert P Yaldizli Ö . Harmonizing definitions for progression independent of relapse activity in multiple sclerosis: a systematic review. JAMA Neurol. (2023) 80:1232–45. doi: 10.1001/jamaneurol.2023.3331 37782515

[21] Abdelhak A Cerono G Sheikhzadeh F Harroud A Ning K Zamecnik CR . Myelin injury precedes axonal injury and symptomatic onset in multiple sclerosis. Nat Med. (2026) 32:362–8. doi: 10.1038/s41591-025-04014-w 41115958 PMC13052337

[22] Dionyssiotis Y Mavrogenis A Trovas G Skarantavos G Papathanasiou J Papagelopoulos P . Bone and soft tissue changes in patients with spinal cord injury and multiple sclerosis. Folia Med (Plovdiv). (2014) 56:237–44. doi: 10.1515/folmed-2015-0002 26444352

[23] Lassmann H . Hypoxia-like tissue injury as a component of multiple sclerosis lesions. J Neurol Sci. (2003) 206:187–91. doi: 10.1016/s0022-510x(02)00421-5 12559509 PMC7130136

[24] Lassmann H . Mechanisms of inflammation induced tissue injury in multiple sclerosis. J Neurol Sci. (2008) 274:45–7. doi: 10.1016/j.jns.2008.04.003 18495163

[25] Jäckle K Zeis T Schaeren-Wiemers N Junker A van der Meer F Kramann N . Molecular signature of slowly expanding lesions in progressive multiple sclerosis. Brain. (2020) 143:2073–88. doi: 10.1093/brain/awaa158 32577755

[26] Maggi P Kuhle J Schädelin S van der Meer F Weigel M Galbusera R . Chronic white matter inflammation and serum neurofilament levels in multiple sclerosis. Neurology. (2021) 97:e543–53. doi: 10.1212/wnl.0000000000012326 34088875 PMC8424501

[27] Bagnato F Mordin M Greene N Mahida S van Wingerden J . Associations between chronic active lesions and clinical outcomes in multiple sclerosis: a systematic literature review. J Managed Care Specialty Pharm. (2025) 31:694–721. doi: 10.18553/jmcp.2025.24294 40357663 PMC12204335

[28] Breville G Benkhoucha M Rezk A Tran NL Senoner I Bar-Or A . Pro-inflammatory c-Met(+) CD4 T cells in multiple sclerosis. Ann Neurol. (2026) 99:261–73. doi: 10.1002/ana.78035 PMC12946607 41002109

[29] Brummer T Zipp F Bittner S . T cell-neuron interaction in inflammatory and progressive multiple sclerosis biology. Curr Opin Neurobiol. (2022) 75:102588. doi: 10.1016/j.conb.2022.102588 35732103

[30] Kunkl M Amormino C Tedeschi V Fiorillo MT Tuosto L . Astrocytes and inflammatory T helper cells: a dangerous liaison in multiple sclerosis. Front Immunol. (2022) 13:824411. doi: 10.3389/fimmu.2022.824411 35211120 PMC8860818

[31] Inglese M Benedetti B Filippi M . The relation between MRI measures of inflammation and neurodegeneration in multiple sclerosis. J Neurol Sci. (2005) 233:15–9. doi: 10.1016/j.jns.2005.03.001 15949493

[32] Sinnecker T Schädelin S Benkert P Ruberte E Amann M Lieb JM . Brain atrophy measurement over a MRI scanner change in multiple sclerosis. NeuroImage Clin. (2022) 36:103148. doi: 10.1016/j.nicl.2022.103148 36007437 PMC9424626

[33] Snoussi H Cohen-Adad J Combès B Bannier É Tounekti S Kerbrat A . Effectiveness of regional diffusion MRI measures in distinguishing multiple sclerosis abnormalities within the cervical spinal cord. Brain Behav. (2023) 13:e3159. doi: 10.1002/brb3.3159 37775975 PMC10636413

[34] Absinta M Sati P Masuzzo F Nair G Sethi V Kolb H . Association of chronic active multiple sclerosis lesions with disability *in vivo*. JAMA Neurol. (2019) 76:1474–83. doi: 10.1001/jamaneurol.2019.2399 31403674 PMC6692692

[35] Hemond CC Gaitán MI Absinta M Reich DS . New imaging markers in multiple sclerosis and related disorders: smoldering inflammation and the central vein sign. Neuroimaging Clin N Am. (2024) 34:359–73. doi: 10.1016/j.nic.2024.03.004 38942521 PMC11213979

[36] Mistry N Hobart J Rog D Muhlert N Mathews J Baker D . Reconciling lesions, relapses and smouldering associated worsening: a unifying model for multiple sclerosis pathogenesis. Mult Scler Relat Disord. (2024) 88:105706. doi: 10.1016/j.msard.2024.105706 38880031

[37] Naval-Baudin P Pons-Escoda A Camins À Arroyo P Viveros M Castell J . Deeply 3D-T1-TFE hypointense voxels are characteristic of phase-rim lesions in multiple sclerosis. Eur Radiol. (2024) 34:1337–45. doi: 10.1007/s00330-023-09784-w 37278854 PMC10853299

[38] Weber CE Wittayer M Kraemer M Dabringhaus A Bail K Platten M . Long-term dynamics of multiple sclerosis iron rim lesions. Mult Scler Relat Disord. (2022) 57:103340. doi: 10.1016/j.msard.2021.103340 35158450

[39] MacMillan EL Russell-Schulz B Alejo G Harp C Cameron B Winger R . Longitudinal magnetic resonance spectroscopy study of metabolite changes over 2 years in relapsing and primary progressive multiple sclerosis treated with ocrelizumab. Mult Scler. (2026), 13524585261456964. doi: 10.1177/13524585261456964 42376811 PMC13646435

[40] Missaglia M Filippi M Esposito F Giordano A . The hypoxia-inflammation cycle as a key mechanism of smoldering inflammation and progression in multiple sclerosis. Acta Neuropathol. (2026) 151. doi: 10.1007/s00401-026-03021-6 42047854

[41] Askari H Rabiei F Lohrasbi F Ghadir S Mehdipour Arbastan A Ghasemi-Kasman M . AMP-activated protein kinase as a mediator of mitochondrial dysfunction of multiple sclerosis in animal models: a systematic review. J Cell Physiol. (2024) 239:e31230. doi: 10.1002/jcp.31230 38403972

[42] de la Rubia Ortí JE Roig-Soriano A Carrera-Juliá S Castelló-Guillen A MaChado M García-Villalba R . Impact of the combination of epigallocatechin gallate and ellagic acid supplemented with ketone bodies on energetic restoration of mitochondrial dysfunction and metabolic inefficiencies in patients with multiple sclerosis: a review. Int J Mol Sci. (2026) 27. doi: 10.3390/ijms27052168 41828397 PMC12985127

[43] Wang PF Jiang F Zeng QM Yin WF Hu YZ Li Q . Mitochondrial and metabolic dysfunction of peripheral immune cells in multiple sclerosis. J Neuroinflamm. (2024) 21:28. doi: 10.1186/s12974-024-03016-8 38243312 PMC10799425

[44] Garg N Reddel SW Miller DH Chataway J Riminton DS Barnett Y . The corpus callosum in the diagnosis of multiple sclerosis and other CNS demyelinating and inflammatory diseases. J Neurol Neurosurg Psychiatry. (2015) 86:1374–82. doi: 10.1136/jnnp-2014-309649 25857658

[45] Suanprasert N Taylor BV Klein CJ Roforth MM Karam C Keegan BM . Polyneuropathies and chronic inflammatory demyelinating polyradiculoneuropathy in multiple sclerosis. Mult Scler Relat Disord. (2019) 30:284–90. doi: 10.1212/wnl.84.14_supplement.s42.001 30870805

[46] Martínez-Pinilla E Rubio-Sardón N Fernández-García G Villar-Conde S Menéndez-Pérez C Tolivia J . Apolipoprotein D expression dynamics during cuprizone-induced demyelination and remyelination in a mouse model of multiple sclerosis. Int J Mol Sci. (2025) 26. doi: 10.3390/ijms26178692 40943611 PMC12429407

[47] Tanaka A Anada K Yasue M Honda T Nakamura H Murayama T . Ceramide kinase knockout ameliorates multiple sclerosis-like behaviors and demyelination in cuprizone-treated mice. Life Sci. (2022) 296:120446. doi: 10.1016/j.lfs.2022.120446 35245521

[48] Wang D Zhang T Shao Q Wu X Zhao X Zhang H . GSDME-mediated pyroptosis in microglia exacerbates demyelination and neuroinflammation in multiple sclerosis: insights from humans and cuprizone-induced demyelination model mice. Cell Death Differ. (2025) 32:2368–83. doi: 10.1038/s41418-025-01537-0 40555745 PMC12669246

[49] Battistini L Selmaj K Kowal C Ohmen J Modlin RL Raine CS . Multiple sclerosis: limited diversity of the V delta 2-J delta 3 T-cell receptor in chronic active lesions. Ann Neurol. (1995) 37:198–203. doi: 10.1002/ana.410370210 7847861

[50] Park C Ponath G Levine-Ritterman M Bull E Swanson EC De Jager PL . The landscape of myeloid and astrocyte phenotypes in acute multiple sclerosis lesions. Acta Neuropathol Commun. (2019) 7:130. doi: 10.1186/s40478-019-0779-2 31405387 PMC6689891

[51] Zeinstra E Wilczak N De Keyser J . Reactive astrocytes in chronic active lesions of multiple sclerosis express co-stimulatory molecules B7-1 and B7-2. J Neuroimmunol. (2003) 135:166–71. doi: 10.1016/s0165-5728(02)00462-9 12576238

[52] Zettl UK Kuhlmann T Brück W . Bcl-2 expressing T lymphocytes in multiple sclerosis lesions. Neuropathol Appl Neurobiol. (1998) 24:202–8. doi: 10.1046/j.1365-2990.1998.00110.x 9717185

[53] Kiljan S Preziosa P Jonkman LE van de Berg WD Twisk J Pouwels PJ . Cortical axonal loss is associated with both gray matter demyelination and white matter tract pathology in progressive multiple sclerosis: Evidence from a combined MRI-histopathology study. Mult Scler. (2021) 27:380–90. doi: 10.1177/1352458520918978 32390507 PMC7897796

[54] Klistorner S Barnett MH Parratt J Yiannikas C Klistorner A . Examining the relative contribution of slow-burning inflammation and chronic demyelination to axonal damage in chronic multiple sclerosis lesions. Mult Scler Relat Disord. (2024) 90:105828. doi: 10.1016/j.msard.2024.105828 39208570

[55] McGavern DB Murray PD Rivera-Quiñones C Schmelzer JD Low PA Rodriguez M . Axonal loss results in spinal cord atrophy, electrophysiological abnormalities and neurological deficits following demyelination in a chronic inflammatory model of multiple sclerosis. Brain. (2000) 123:519–31. doi: 10.1093/brain/123.3.519 10686175 PMC5444460

[56] Sedel F Bernard D Mock DM Tourbah A . Targeting demyelination and virtual hypoxia with high-dose biotin as a treatment for progressive multiple sclerosis. Neuropharmacology. (2016) 110:644–53. doi: 10.1016/j.neuropharm.2015.08.028 26327679

[57] Tallantyre EC Bø L Al-Rawashdeh O Owens T Polman CH Lowe JS . Clinico-pathological evidence that axonal loss underlies disability in progressive multiple sclerosis. Mult Scler. (2010) 16:406–11. doi: 10.1177/1352458510364992 20215480

[58] Abo-Elsoud RAA Ali EA Al-Gholam MA Rizk MS Elseadawy RSA Ameen O . Pirfenidone mitigates demyelination and electrophysiological alterations in multiple sclerosis: Targeting NF-κB, sirt1, and neurotrophic genes. Naunyn Schmiedebergs Arch Pharmacol. (2025) 398:4019–36. doi: 10.1007/s00210-024-03496-8 39404841 PMC11978544

[59] Denaroso GE Smith Z Angeliu CG Cheli VT Wang C Paez PM . Deletion of voltage-gated calcium channels in astrocytes decreases neuroinflammation and demyelination in a murine model of multiple sclerosis. J Neuroinflamm. (2023) 20:263. doi: 10.1186/s12974-023-02948-x 37964385 PMC10644533

[60] Farzan M Saberi-Rounkian M Asadi-Rizi A Heidari Z Farzan M Fathi M . The emerging role of the microglia triggering receptor expressed on myeloid cells (TREM) 2 in multiple sclerosis. Exp Neurol. (2025) 384:115071. doi: 10.1016/j.expneurol.2024.115071 39586397

[61] Guadalupi L Vanni V Balletta S Caioli S De Vito F Fresegna D . Interleukin-9 protects from microglia- and TNF-mediated synaptotoxicity in experimental multiple sclerosis. J Neuroinflamm. (2024) 21:128. doi: 10.1186/s12974-024-03120-9 38745307 PMC11092167

[62] Nuesslein-Hildesheim B Ferrero E Schmid C Huck C Smith P Tisserand S . Remibrutinib (LOU064) inhibits neuroinflammation driven by B cells and myeloid cells in preclinical models of multiple sclerosis. J Neuroinflamm. (2023) 20:194. doi: 10.1186/s12974-023-02877-9 37633912 PMC10463946

[63] Hofmann A Krajnc N Dal-Bianco A Riedl CJ Zrzavy T Lerma-Martin C . Myeloid cell iron uptake pathways and paramagnetic rim formation in multiple sclerosis. Acta Neuropathol. (2023) 146:707–24. doi: 10.1007/s00401-023-02627-4 37715818 PMC10564819

[64] Drake SS Zaman A Simas T Fournier AE . Comparing RNA-sequencing datasets from astrocytes, oligodendrocytes, and microglia in multiple sclerosis identifies novel dysregulated genes relevant to inflammation and myelination. WIREs Mech Dis. (2023) 15:e1594. doi: 10.1002/wsbm.1594 36600404

[65] Farez MF Correale J . Sphingosine 1-phosphate signaling in astrocytes: Implications for progressive multiple sclerosis. J Neurol Sci. (2016) 361:60–5. doi: 10.1016/j.jns.2015.12.022 26810518

[66] Kamermans A Planting KE Jalink K van Horssen J de Vries HE . Reactive astrocytes in multiple sclerosis impair neuronal outgrowth through TRPM7-mediated chondroitin sulfate proteoglycan production. Glia. (2019) 67:68–77. doi: 10.1002/glia.23526 30453391 PMC6587975

[67] Kerkering J Muinjonov B Rosiewicz KS Diecke S Biese C Schiweck J . iPSC-derived reactive astrocytes from patients with multiple sclerosis protect cocultured neurons in inflammatory conditions. J Clin Invest. (2023) 133. doi: 10.1172/jci164637 37219933 PMC10313373

[68] Kazimuddin HF Wang J Toubasi AA Hernandez B Sun L Vinarsky T . Paramagnetic rim lesions in early multiple sclerosis: a 7 Tesla imaging study. Brain Commun. (2025) 7:fcaf215. doi: 10.1093/braincomms/fcaf215 40585811 PMC12203533

[69] Krajnc N Schmidbauer V Leinkauf J Haider L Bsteh G Kasprian G . Paramagnetic rim lesions lead to pronounced diffuse periplaque white matter damage in multiple sclerosis. Mult Scler. (2023) 29:1406–17. doi: 10.1177/13524585231197954 37712486 PMC10580674

[70] Preziosa P Pagani E Meani A Margoni M Rubin M Esposito F . Soma and neurite density abnormalities of paramagnetic rim lesions and core-sign lesions in multiple sclerosis. J Neurol. (2025) 272:145. doi: 10.1007/s00415-025-12887-7 39812706

[71] Rocca MA Preziosa P Filippi M . Juxtacortical paramagnetic rim: A new MRI marker to characterize focal cortical pathology in multiple sclerosis? Neurology. (2024) 102:e208085. doi: 10.1212/wnl.0000000000208085 38165304

[72] Zhu Z Zhang Y Li C Guo W Chen Z Chen W . Paramagnetic rim lesions as a biomarker to discriminate between multiple sclerosis and cerebral small vessel disease. Front Neurol. (2024) 15:1429698. doi: 10.3389/fneur.2024.1429698 39081339 PMC11286476

[73] Asadpour S Mazdeh M Karimi J Khodadadi I Shafiee G . Vitamin D3 supplementation modulates inflammatory protein CHI3L1/YKL-40 and oxidative stress status in multiple sclerosis. Neuropsychopharmacol Rep. (2025) 45:e70076. doi: 10.1002/npr2.70076 41277171 PMC12641099

[74] Tonev D Momchilova A . Oxidative stress and the nuclear factor erythroid 2-related factor 2 (Nrf2) pathway in multiple sclerosis: focus on certain exogenous and endogenous nrf2 activators and therapeutic plasma exchange modulation. Int J Mol Sci. (2023) 24. doi: 10.3390/ijms242417223 38139050 PMC10743556

[75] Luoqian J Yang W Ding X Tuo QZ Xiang Z Zheng Z . Ferroptosis promotes T-cell activation-induced neurodegeneration in multiple sclerosis. Cell Mol Immunol. (2022) 19:913–24. doi: 10.1038/s41423-022-00883-0 35676325 PMC9338013

[76] Mishra MK Wang J Mirzaei R Chan R Melo H Zhang P . A distinct hibiscus sabdariffa extract prevents iron neurotoxicity, a driver of multiple sclerosis pathology. Cells. (2022) 11. doi: 10.3390/cells11030440 35159249 PMC8834068

[77] Riedl CJ Bormann D Steinmaurer A Novak A Testa G Poldlehner E . Inflammation alters myeloid cell and oligodendroglial iron-handling in multiple sclerosis. Acta Neuropathol Commun. (2025) 13:124. doi: 10.1186/s40478-025-02020-0 40468400 PMC12135419

[78] Choi S Lake S Harrison DM . Evaluation of the blood-brain barrier, demyelination, and neurodegeneration in paramagnetic rim lesions in multiple sclerosis on 7 tesla MRI. J Magnetic Resonance Imaging JMRI. (2024) 59:941–51. doi: 10.1002/jmri.28847 37276054 PMC10754232

[79] Al-Radaideh A El-Haj N Hijjawi N . Iron deposition and atrophy in cerebral grey matter and their possible association with serum iron in relapsing-remitting multiple sclerosis. Clin Imaging. (2021) 69:238–42. doi: 10.1016/j.clinimag.2020.09.006 32977196

[80] Oliveira SR Kallaur AP Lopes J Colado Simão AN Reiche EM de Almeida ERD . Insulin resistance, atherogenicity, and iron metabolism in multiple sclerosis with and without depression: Associations with inflammatory and oxidative stress biomarkers and uric acid. Psychiatry Res. (2017) 250:113–20. doi: 10.1016/j.psychres.2016.12.039 28152396

[81] Gillen KM Mubarak M Nguyen TD Pitt D . Significance and *in vivo* detection of iron-laden microglia in white matter multiple sclerosis lesions. Front Immunol. (2018) 9:255. doi: 10.3389/fimmu.2018.00255 29515576 PMC5826076

[82] Hsiao CC Fransen NL van den Bosch AMR Brandwijk KIM Huitinga I Hamann J . White matter lesions in multiple sclerosis are enriched for CD20(dim) CD8(+) tissue-resident memory T cells. Eur J Immunol. (2021) 51:483–6. doi: 10.1002/eji.202048665 32949467

[83] Ahmed SM Fransen NL Touil H Michailidou I Huitinga I Gommerman JL . Accumulation of meningeal lymphocytes correlates with white matter lesion activity in progressive multiple sclerosis. JCI Insight. (2022) 7. doi: 10.1172/jci.insight.151683 35104246 PMC8983127

[84] Londoño AC Mora CA . Role of CXCL13 in the formation of the meningeal tertiary lymphoid organ in multiple sclerosis. F1000Res. (2018) 7:514. doi: 10.12688/f1000research.14556.3 30345018 PMC6171727

[85] Londoño AC Mora CA . High efficacy therapy to prevent the formation of meningeal tertiary lymphoid organs after CXCL13 index screening in early multiple sclerosis. Front Neurosci. (2025) 19:1558810. doi: 10.3389/fnins.2025.1558810 40165834 PMC11955623

[86] Magliozzi R Howell OW Durrenberger P Aricò E James R Cruciani C . Meningeal inflammation changes the balance of TNF signalling in cortical grey matter in multiple sclerosis. J Neuroinflamm. (2019) 16:259. doi: 10.1186/s12974-019-1650-x 31810488 PMC6898969

[87] Reali C Magliozzi R Roncaroli F Nicholas R Howell OW Reynolds R . B cell rich meningeal inflammation associates with increased spinal cord pathology in multiple sclerosis. Brain Pathol. (2020) 30:779–93. doi: 10.1111/bpa.12841 32243032 PMC8018043

[88] Zurawski J Tuncer A Profant MR Wang J Green M Park Y . ODC1 restricts meningeal B cell age-associated-like phenotype and function in multiple sclerosis: A human and experimental study. Proc Natl Acad Sci USA. (2026) 123:e2526147123. doi: 10.1073/pnas.2526147123 41824499 PMC12993946

[89] Bajrami A Tamanti A Peloso A Ziccardi S Guandalini M Calderone M . Ocrelizumab reduces cortical and deep grey matter loss compared to the S1P-receptor modulator in multiple sclerosis. J Neurol. (2024) 271:2149–58. doi: 10.1007/s00415-023-12179-y 38289534 PMC11055717

[90] Cortese R Battaglini M Prados F Gentile G Luchetti L Bianchi A . Grey matter atrophy and its relationship with white matter lesions in patients with myelin oligodendrocyte glycoprotein antibody-associated disease, aquaporin-4 antibody-positive neuromyelitis optica spectrum disorder, and multiple sclerosis. Ann Neurol. (2024) 96:276–88. doi: 10.1002/ana.26951 38780377

[91] Kutzelnigg A Lucchinetti CF Stadelmann C Brück W Rauschka H Bergmann M . Cortical demyelination and diffuse white matter injury in multiple sclerosis. Brain. (2005) 128:2705–12. doi: 10.1093/brain/awh641 16230320

[92] Platten M Ouellette R Herranz E Barletta V Treaba CA Mainero C . Cortical and white matter lesion topology influences focal corpus callosum atrophy in multiple sclerosis. J Neuroimaging. (2022) 32:471–9. doi: 10.1111/jon.12977 35165979 PMC9305945

[93] Okutan B Frederiksen JL Houen G Sellebjerg F Kyllesbech C Magyari M . Subcortical plaques and inflammation reflect cortical and meningeal pathologies in progressive multiple sclerosis. Brain Pathol. (2025) 35:e13314. doi: 10.1111/bpa.13314 39460678 PMC11961212

[94] Bevan RJ Evans R Griffiths L Watkins LM Rees MI Magliozzi R . Meningeal inflammation and cortical demyelination in acute multiple sclerosis. Ann Neurol. (2018) 84:829–42. doi: 10.1002/ana.25365 30362156

[95] James RE Schalks R Browne E Eleftheriadou I Munoz CP Mazarakis ND . Persistent elevation of intrathecal pro-inflammatory cytokines leads to multiple sclerosis-like cortical demyelination and neurodegeneration. Acta Neuropathol Commun. (2020) 8:66. doi: 10.1186/s40478-020-00938-1 32398070 PMC7218553

[96] Pikor NB Prat A Bar-Or A Gommerman JL . Meningeal tertiary lymphoid tissues and multiple sclerosis: A gathering place for diverse types of immune cells during CNS autoimmunity. Front Immunol. (2015) 6:657. doi: 10.3389/fimmu.2015.00657 26793195 PMC4710700

[97] Najm L Chomyk A Cyncynatus K Davalos D Mahajan KR Trapp BD . Demyelinated lesion associated compartmental inflammation in progressive multiple sclerosis brains. Acta Neuropathol Commun. (2025) 13:204. doi: 10.1186/s40478-025-02122-9 41024168 PMC12482250

[98] van Wageningen TA Vlaar E Kooij G Jongenelen CAM Geurts JJG van Dam AM . Regulation of microglial TMEM119 and P2RY12 immunoreactivity in multiple sclerosis white and grey matter lesions is dependent on their inflammatory environment. Acta Neuropathol Commun. (2019) 7:206. doi: 10.1186/s40478-019-0850-z 31829283 PMC6907356

[99] Cabezas M Oliver A Roura E Freixenet J Vilanova JC Ramió-Torrentà L . Automatic multiple sclerosis lesion detection in brain MRI by FLAIR thresholding. Comput Methods Programs BioMed. (2014) 115:147–61. doi: 10.1016/j.cmpb.2014.04.006 24813718

[100] Iancheva D Trenova A Mantarova S Terziyski K . Structural and functional MRI techniques in multiple sclerosis related cognitive dysfunction. Folia Med (Plovdiv). (2018) 60:505–11. doi: 10.2478/folmed-2018-0031 31188776

[101] Evans R Watkins LM Hawkins K Santiago G Demetriou C Naughton M . Complement activation and increased anaphylatoxin receptor expression are associated with cortical grey matter lesions and the compartmentalised inflammatory response of multiple sclerosis. Front Cell Neurosci. (2023) 17:1094106. doi: 10.3389/fncel.2023.1094106 37032838 PMC10073739

[102] Zhu Q Yan Z Shi Z Luo D Ding S Chen X . Increased cortical lesion load contributed to pathological changes beyond focal lesion in cortical gray matter of multiple sclerosis: a diffusion kurtosis imaging analysis. Cereb Cortex. (2023) 33:10867–76. doi: 10.1093/cercor/bhad332 37718158

[103] Duan J Lv A Guo Z Liu Q Tian C Yang Y . CX3CR1(+)/UCHL1(+) microglial extracellular vesicles in blood: a potential biomarker for multiple sclerosis. J Neuroinflamm. (2024) 21:254. doi: 10.1186/s12974-024-03243-z 39385200 PMC11465848

[104] Ju WY Wang Q Song LJ Ding ZB Li XH Kumar G . Drug-induced microglial phagocytosis in multiple sclerosis and experimental autoimmune encephalomyelitis and the underlying mechanisms. Mol Biol Rep. (2023) 50:749–59. doi: 10.1007/s11033-022-07968-z 36309614

[105] Yan W Jianhong W Ping G . NLRC5-mediated epigenetic and proteomic regulation of microglial panoptosis drives neuroinflammation in multiple sclerosis. Mol Neurobiol. (2025) 63:194. doi: 10.1007/s12035-025-05365-8 41313522

[106] Airas L Nylund M Rissanen E . Evaluation of microglial activation in multiple sclerosis patients using positron emission tomography. Front Neurol. (2018) 9:181. doi: 10.3389/fneur.2018.00181 29632509 PMC5879102

[107] Kara F Neyal N Kamykowski MG Schwarz CG Kendall-Thomas J Morrison HA . Decoding thalamic glial interplay in multiple sclerosis through proton magnetic resonance spectroscopy and positron emission tomography. Int J Mol Sci. (2025) 26. doi: 10.3390/ijms26178656 40943577 PMC12428814

[108] Matías-Guiu JA Cabrera-Martín MN Pytel V Montero P Carreras JL Matías-Guiu J . Amyloid positron emission tomography in multiple sclerosis: Between amyloid deposition and myelin damage. Ann Neurol. (2020) 87:988. doi: 10.1002/ana.25759 32356353

[109] Guido G Preziosa P Filippi M Rocca MA . TSPO PET in multiple sclerosis: Emerging insights into pathophysiology, prognosis, and treatment monitoring. Mult Scler Relat Disord. (2025) 100:106546. doi: 10.1016/j.msard.2025.106546 40450829

[110] Nylund M Lehto J Matilainen M Rajander J Wahlroos S Sucksdorff M . Longitudinal accumulation of glial activation measured by TSPO-PET predicts later brain atrophy in multiple sclerosis. J Neuroinflamm. (2025) 22:200. doi: 10.1186/s12974-025-03519-y 40775641 PMC12333212

[111] Sjöros T Saraste M Matilainen M Nylund M Koivumäki M Kuhle J . Serum glial fibrillary acid protein associates with TSPO-expressing lesions in multiple sclerosis brain. Ther Adv Neurol Disord. (2025) 18:17562864251352998. doi: 10.1177/17562864251352998 40756531 PMC12314351

[112] Tang S Harrison DM Bardhoshi A Cureton R Yan X Parcon PA . Cyclooxygenase-1 and cyclooxygenase-2 densities measured using positron emission tomography are not altered in the brains of individuals with stable multiple sclerosis. J Cereb Blood Flow Metab. (2025) 45:1417–27. doi: 10.1177/0271678x251332490 40367389 PMC12078256

[113] Elliott C Wolinsky JS Hauser SL Kappos L Barkhof F Bernasconi C . Slowly expanding/evolving lesions as a magnetic resonance imaging marker of chronic active multiple sclerosis lesions. Mult Scler. (2019) 25:1915–25. doi: 10.1177/1352458518814117 30566027 PMC6876256

[114] Fadda G Banwell B Elliott C Fetco D Arnold DL Waters P . Slowly expanding lesions differentiate pediatric multiple sclerosis from myelin oligodendrocyte glycoprotein antibody disease. Ann Neurol. (2024) 96:1086–91. doi: 10.1002/ana.27066 39243229

[115] Messmer ML Salapa HE Popescu BF Levin MC . RNA binding protein dysfunction links smoldering/slowly expanding lesions to neurodegeneration in multiple sclerosis. Ann Neurol. (2025) 97:313–28. doi: 10.1002/ana.27114 39422285

[116] Dal-Bianco A Grabner G Kronnerwetter C Weber M Kornek B Kasprian G . Long-term evolution of multiple sclerosis iron rim lesions in 7 T MRI. Brain. (2021) 144:833–47. doi: 10.1093/brain/awaa436 33484118

[117] Marcille M Hurtado Rúa S Tyshkov C Jaywant A Comunale J Kaunzner UW . Disease correlates of rim lesions on quantitative susceptibility mapping in multiple sclerosis. Sci Rep. (2022) 12:4411. doi: 10.1038/s41598-022-08477-6 35292734 PMC8924224

[118] Reeves JA Bartnik A Jakimovski D Mohebbi M Bergsland N Salman F . Associations between paramagnetic rim lesion evolution and clinical and radiologic disease progression in persons with multiple sclerosis. Neurology. (2024) 103:e210004. doi: 10.1212/wnl.0000000000210004 39447104 PMC11509899

[119] Vavasour I Elliott C Arnold DL Gaetano L Clayton D Magon S . Presence of slowly expanding lesions in multiple sclerosis predicts progressive demyelination within lesions and normal-appearing tissue over time. Mult Scler. (2025) 31:418–32. doi: 10.1177/13524585251316519 39950257 PMC11956371

[120] Calvi A Carrasco FP Tur C Chard DT Stutters J De Angelis F . Association of slowly expanding lesions on MRI with disability in people with secondary progressive multiple sclerosis. Neurology. (2022) 98:e1783–93. doi: 10.1212/wnl.0000000000200144 35277438

[121] Elliott C Rudko DA Arnold DL Fetco D Elkady AM Araujo D . Lesion-level correspondence and longitudinal properties of paramagnetic rim and slowly expanding lesions in multiple sclerosis. Mult Scler. (2023) 29:680–90. doi: 10.1177/13524585231162262 37036134 PMC10176750

[122] Huang W Sweeney EM Kaunzner UW Wang Y Gauthier SA Nguyen TD . Quantitative susceptibility mapping versus phase imaging to identify multiple sclerosis iron rim lesions with demyelination. J Neuroimaging. (2022) 32:667–75. doi: 10.1111/jon.12987 35262241 PMC9308704

[123] Gillen KM Nguyen TD Dimov A Kovanlikaya I Luu HM Demmon E . Quantitative susceptibility mapping is more sensitive and specific than phase imaging in detecting chronic active multiple sclerosis lesion rims: pathological validation. Brain Commun. (2025) 7:fcaf011. doi: 10.1093/braincomms/fcaf011 39916751 PMC11800486

[124] Cagol A Schaedelin S Ocampo-Pineda M Benkert P Melie-Garcia L Luchetti L . Comparative effectiveness of teriflunomide and ocrelizumab on smoldering activity in multiple sclerosis: an observational study in the Swiss Multiple Sclerosis Cohort. J Neurol. (2025) 272:491. doi: 10.1007/s00415-025-13221-x 40603656 PMC12222313

[125] Shi Z Wang H Yan Z Tang Y Moccia M Nie L . T1-based stratification of paramagnetic rim lesions in multiple sclerosis: Evidence of microstructural heterogeneity and clinical implications. Mult Scler. (2025) 31:1651–63. doi: 10.1177/13524585251389809 41208452

[126] Polvinen E Matilainen M Nylund M Sucksdorff M Airas LM . TSPO-detectable chronic active lesions predict disease progression in multiple sclerosis. Neurol Neuroimmunol Neuroinflamm. (2023) 10. doi: 10.1212/nxi.0000000000200133 37349108 PMC10291892

[127] Vanheule E Cambron M Dobai A Casselman JW . Rim lesions in MS at 3T: clinical correlation and possible radiological alternatives for daily practice at lower field strength. J Neuroradiol. (2024) 51:101165. doi: 10.1016/j.neurad.2023.10.010 37907156

[128] Klotz L Smolders J Lehto J Matilainen M Lütje L Buchholz L . Broad rim lesions are a new pathological and imaging biomarker for rapid disease progression in multiple sclerosis. Nat Med. (2025) 31:2016–26. doi: 10.1038/s41591-025-03625-7 40301560 PMC12176629

[129] Deh K Ponath GD Molvi Z Parel GT Gillen KM Zhang S . Magnetic susceptibility increases as diamagnetic molecules breakdown: Myelin digestion during multiple sclerosis lesion formation contributes to increase on QSM. J Magn Reson Imaging. (2018) 48:1281–7. doi: 10.1002/jmri.25997 29517817 PMC6129234

[130] Gillen KM Mubarak M Park C Ponath G Zhang S Dimov A . QSM is an imaging biomarker for chronic glial activation in multiple sclerosis lesions. Ann Clin Transl Neurol. (2021) 8:877–86. doi: 10.1002/acn3.51338 33704933 PMC8045922

[131] Reeves JA Mohebbi M Zivadinov R Bergsland N Dwyer MG Salman F . Reliability of paramagnetic rim lesion classification on quantitative susceptibility mapping (QSM) in people with multiple sclerosis: Single-site experience and systematic review. Mult Scler Relat Disord. (2023) 79:104968. doi: 10.1016/j.msard.2023.104968 37716210 PMC11092095

[132] Wang H Tang Y Yan Z Zhu Q Nie L Wei H . Subvoxel quantitative susceptibility mapping of chronic white matter lesions: Enhancing lesion heterogeneity characterization and providing complementary biomarkers for myelin integrity in multiple sclerosis. Mult Scler Relat Disord. (2025) 103:106698. doi: 10.1016/j.msard.2025.106698 40850229

[133] Zhang Y Gauthier SA Gupta A Comunale J Chia-Yi Chiang G Zhou D . Longitudinal change in magnetic susceptibility of new enhanced multiple sclerosis (MS) lesions measured on serial quantitative susceptibility mapping (QSM). J Magn Reson Imaging. (2016) 44:426–33. doi: 10.1002/jmri.25144 26800367 PMC4946979

[134] Zhu Z Naji N Esfahani JH Snyder J Seres P Emery DJ . MR susceptibility separation for quantifying lesion paramagnetic and diamagnetic evolution in relapsing-remitting multiple sclerosis. J Magn Reson Imaging. (2024) 60:1867–79. doi: 10.1002/jmri.29266 38308397

[135] Donatelli G Cecchi P Migaleddu G Cencini M Frumento P D'Amelio C . Quantitative T1 mapping detects blood-brain barrier breakdown in apparently non-enhancing multiple sclerosis lesions. NeuroImage Clin. (2023) 40:103509. doi: 10.1016/j.nicl.2023.103509 37717382 PMC10514220

[136] Hansen CE Kamermans A Mol K Berve K Rodriguez-Mogeda C Fung WK . Inflammation-induced TRPV4 channels exacerbate blood-brain barrier dysfunction in multiple sclerosis. J Neuroinflamm. (2024) 21:72. doi: 10.1186/s12974-024-03069-9 38521959 PMC10960997

[137] Luo Y Yang H Wan Y Yang S Wu J Chen S . Endothelial ETS1 inhibition exacerbate blood-brain barrier dysfunction in multiple sclerosis through inducing endothelial-to-mesenchymal transition. Cell Death Dis. (2022) 13:462. doi: 10.1038/s41419-022-04888-5 35568723 PMC9107459

[138] Nowaczewska-Kuchta A Ksiazek-Winiarek D Glabinski A . Interaction between neutrophils and elements of the blood-brain barrier in the context of multiple sclerosis and ischemic stroke. Int J Mol Sci. (2025) 26. doi: 10.3390/ijms26094437 40362673 PMC12072651

[139] Wang Y Ma X Qiao Z Zhang X Ji J Zhang S . Electroacupuncture alleviates blood-brain barrier disruption and neuroinflammation via astrocytic MC4R in a mouse model of multiple sclerosis. J Neuroinflamm. (2025) 23:40. doi: 10.1186/s12974-025-03667-1 41454349 PMC12849424

[140] Elliott C Belachew S Wolinsky JS Hauser SL Kappos L Barkhof F . Chronic white matter lesion activity predicts clinical progression in primary progressive multiple sclerosis. Brain A J Neurol. (2019) 142:2787–99. doi: 10.1093/brain/awz212 31497864 PMC6736181

[141] Preziosa P Pagani E Meani A Moiola L Rodegher M Filippi M . Slowly expanding lesions predict 9-year multiple sclerosis disease progression. Neurology(R) neuroimmunology Neuroinflamm. (2022) 9. doi: 10.1212/nxi.0000000000001139 35105685 PMC8808355

[142] Tozlu C Jamison K Nguyen T Zinger N Kaunzner U Pandya S . Structural disconnectivity from paramagnetic rim lesions is related to disability in multiple sclerosis. Brain Behav. (2021) 11:e2353. doi: 10.1002/brb3.2353 34498432 PMC8553317

[143] Bjartmar C Kidd G Mörk S Rudick R Trapp BD . Neurological disability correlates with spinal cord axonal loss and reduced N-acetyl aspartate in chronic multiple sclerosis patients. Ann Neurol. (2000) 48:893–901. doi: 10.1007/978-88-470-2109-9_3 11117546

[144] Brück W . The pathology of multiple sclerosis is the result of focal inflammatory demyelination with axonal damage. J Neurol. (2005) 252:v3–9. doi: 10.1007/s00415-005-5002-7 16254699

[145] Haider L Zrzavy T Hametner S Höftberger R Bagnato F Grabner G . The topograpy of demyelination and neurodegeneration in the multiple sclerosis brain. Brain. (2016) 139:807–15. doi: 10.1093/brain/awv398 26912645 PMC4766379

[146] Seehusen F Baumgärtner W . Axonal pathology and loss precede demyelination and accompany chronic lesions in a spontaneously occurring animal model of multiple sclerosis. Brain Pathol. (2010) 20:551–9. doi: 10.1111/j.1750-3639.2009.00332.x 19775292 PMC8094816

[147] Camara-Lemarroy C Silva C Gohill J Yong VW Koch M . Serum neurofilament-light and glial fibrillary acidic protein levels in hydroxychloroquine-treated primary progressive multiple sclerosis. Eur J Neurol. (2023) 30:187–94. doi: 10.1111/ene.15588 36214614

[148] Meier S Willemse EAJ Schaedelin S Oechtering J Lorscheider J Melie-Garcia L . Serum glial fibrillary acidic protein compared with neurofilament light chain as a biomarker for disease progression in multiple sclerosis. JAMA Neurol. (2023) 80:287–97. doi: 10.1001/jamaneurol.2022.5250 36745446 PMC10011932

[149] Ulndreaj A Sohaei D Thebault S Pons-Belda OD Fernandez-Uriarte A Campbell C . Quantitation of neurofilament light chain protein in serum and cerebrospinal fluid from patients with multiple sclerosis using the MSD R-PLEX NfL assay. Diagnosis (Berl). (2023) 10:275–80. doi: 10.1515/dx-2022-0125 36788117 PMC10424569

[150] Yik JT Becquart P Gill J Petkau J Traboulsee A Carruthers R . Serum neurofilament light chain correlates with myelin and axonal magnetic resonance imaging markers in multiple sclerosis. Mult Scler Relat Disord. (2022) 57:103366. doi: 10.1016/j.msard.2021.103366 35158472

[151] Reeves JA Mohebbi M Wicks T Salman F Bartnik A Jakimovski D . Paramagnetic rim lesions predict greater long-term relapse rates and clinical progression over 10 years. Mult Scler. (2024) 30:535–45. doi: 10.1177/13524585241229956 38366920 PMC11009059

[152] Harrison DM Roy S Oh J Izbudak I Pham D Courtney S . Association of cortical lesion burden on 7-T magnetic resonance imaging with cognition and disability in multiple sclerosis. JAMA Neurol. (2015) 72:1004–12. doi: 10.1001/jamaneurol.2015.1241 26192316 PMC4620027

[153] Zurawski J Tauhid S Healy BC Chu R Houtchens MK Jalkh Y . Cladribine is associated with stable cortical gray matter lesion burden in multiple sclerosis: A 7T MRI study. J Neuroimaging. (2025) 35:e70032. doi: 10.1111/jon.70032 40103263

[154] Balasa R Barcutean L Balasa A Motataianu A Roman-Filip C Manu D . The action of TH17 cells on blood brain barrier in multiple sclerosis and experimental autoimmune encephalomyelitis. Hum Immunol. (2020) 81:237–43. doi: 10.1016/j.humimm.2020.02.009 32122685

[155] Sorosina M Peroni S Mascia E Santoro S Osiceanu AM Ferrè L . Involvement of NINJ2 protein in inflammation and blood-brain barrier transmigration of monocytes in multiple sclerosis. Genes (Basel). (2022) 13. doi: 10.3390/genes13111946 36360183 PMC9690398

[156] Zhang L Tang S Ma Y Liu J Monnier P Li H . RGMa participates in the blood-brain barrier dysfunction through BMP/BMPR/YAP signaling in multiple sclerosis. Front Immunol. (2022) 13:861486. doi: 10.3389/fimmu.2022.861486 35664003 PMC9159795

[157] Meyer-Moock S Feng YS Maeurer M Dippel FW Kohlmann T . Systematic literature review and validity evaluation of the Expanded Disability Status Scale (EDSS) and the Multiple Sclerosis Functional Composite (MSFC) in patients with multiple sclerosis. BMC Neurol. (2014) 14:58. doi: 10.1016/j.jval.2013.08.1560 24666846 PMC3986942

[158] Song X Li D Qiu Z Su S Wu Y Wang J . Correlation between EDSS scores and cervical spinal cord atrophy at 3T MRI in multiple sclerosis: A systematic review and meta-analysis. Mult Scler Relat Disord. (2020) 37:101426. doi: 10.1016/j.msard.2019.101426 32172997

[159] Cacciaguerra L Rocca MA Filippi M . Understanding the pathophysiology and magnetic resonance imaging of multiple sclerosis and neuromyelitis optica spectrum disorders. Korean J Radiol. (2023) 24:1260–83. doi: 10.3348/kjr.2023.0360 38016685 PMC10700997

[160] Manogaran P Hanson JV Olbert ED Egger C Wicki C Gerth-Kahlert C . Optical coherence tomography and magnetic resonance imaging in multiple sclerosis and neuromyelitis optica spectrum disorder. Int J Mol Sci. (2016) 17. doi: 10.3390/ijms17111894 27854301 PMC5133893

[161] Hersh CM Gorritz M Chen CC Tuly R Gu Y Gadkari A . Real-world persistence and adherence of ofatumumab versus oral and injectable disease-modifying therapies in patients with multiple sclerosis. Mult Scler Relat Disord. (2024) 91:105888. doi: 10.1016/j.msard.2024.105888 39388747

[162] Melzer N Meuth SG . Disease-modifying therapy in multiple sclerosis and chronic inflammatory demyelinating polyradiculoneuropathy: Common and divergent current and future strategies. Clin Exp Immunol. (2014) 175:359–72. doi: 10.1111/cei.12195 24032475 PMC3927897

[163] Owens GM Olvey EL Skrepnek GH Pill MW . Perspectives for managed care organizations on the burden of multiple sclerosis and the cost-benefits of disease-modifying therapies. J Manag Care Pharm. (2013) 19:S41–53. doi: 10.18553/jmcp.2013.19.s1.s41 23383732 PMC10438212

[164] Masuda H Mori M Uchida T Uzawa A Ohtani R Kuwabara S . Soluble CD40 ligand contributes to blood-brain barrier breakdown and central nervous system inflammation in multiple sclerosis and neuromyelitis optica spectrum disorder. J Neuroimmunol. (2017) 305:102–7. doi: 10.1016/j.jneuroim.2017.01.024 28284329

[165] Shapiro AM Jack CS Lapierre Y Arbour N Bar-Or A Antel JP . Potential for interferon beta-induced serum antibodies in multiple sclerosis to inhibit endogenous interferon-regulated chemokine/cytokine responses within the central nervous system. Arch Neurol. (2006) 63:1296–9. doi: 10.1001/jama.1951.03670250069029 16966508

[166] Zhang Q Chen Z Zhang K Zhu J Jin T . FGF/FGFR system in the central nervous system demyelinating disease: Recent progress and implications for multiple sclerosis. CNS Neurosci Ther. (2023) 29:1497–511. doi: 10.1111/cns.14176 36924298 PMC10173727

[167] Maggi P Bulcke V Pedrini E Bugli C Sellimi A Wynen M . B cell depletion therapy does not resolve chronic active multiple sclerosis lesions. EBioMedicine. (2023) 94:104701. doi: 10.1016/j.ebiom.2023.104701 37437310 PMC10436266

[168] Baker D Kang AS Giovannoni G Schmierer K . Neutropenia following immune-depletion, notably CD20 targeting, therapies in multiple sclerosis. Mult Scler Relat Disord. (2024) 82:105400. doi: 10.1016/j.msard.2023.105400 38181696

[169] Gelfand JM Cree BAC Hauser SL . Ocrelizumab and other CD20(+) B-cell-depleting therapies in multiple sclerosis. Neurotherapeutics. (2017) 14:835–41. doi: 10.1007/s13311-017-0557-4 28695471 PMC5722762

[170] Margoni M Battistini L Centonze D Furlan R Gasperini C Filippi M . Pharmacodynamics and pharmacokinetics of ublituximab compared with other anti-CD20 monoclonal antibodies for multiple sclerosis treatment. Curr Neuropharmacol. (2026) 24:672–82. doi: 10.2174/011570159x392955250815095236 40873276 PMC13269908

[171] Bellassen L David K Lampert B Sarusi-Portuguez A Tsoory M Lubliner J . The immune receptor SLAMF5 regulates myeloid-cell mediated neuroinflammation in multiple sclerosis. PloS Biol. (2025) 23:e3003373. doi: 10.1371/journal.pbio.3003373 40920820 PMC12431667

[172] Jelcic I Naghavian R Fanaswala I Macnair W Esposito C Calini D . T-bet+ CXCR3+ B cells drive hyperreactive B-T cell interactions in multiple sclerosis. Cell Rep Med. (2025) 6:102027. doi: 10.1016/j.xcrm.2025.102027 40107244 PMC11970401

[173] Kiss MG Mindur JE Yates AG Lee D Fullard JF Anzai A . Interleukin-3 coordinates glial-peripheral immune crosstalk to incite multiple sclerosis. Immunity. (2023) 56:1502–1514.e1508. doi: 10.1016/j.immuni.2023.04.013 37160117 PMC10524830

[174] Zinger N Ponath G Sweeney E Nguyen TD Lo CH Diaz I . Dimethyl fumarate reduces inflammation in chronic active multiple sclerosis lesions. Neurol Neuroimmunol Neuroinflamm. (2022) 9. doi: 10.1212/nxi.0000000000001138 35046083 PMC8771666

[175] Ovchinnikov A Findling O . An overview of pivotal trials and real-world evidence for CD20-depleting therapy in multiple sclerosis : Immunotherapy with rituximab, ocrelizumab, and ofatumumab. Wien Med Wochenschr. (2022) 172:359–64. doi: 10.1007/s10354-022-00939-w 35723820 PMC9208251

[176] Willison AG Hagler R Weise M Elben S Huntemann N Masanneck L . Effects of anti-CD20 antibody therapy on immune cell dynamics in relapsing-remitting multiple sclerosis. Cells. (2025) 14. doi: 10.3390/cells14070552 40214505 PMC11988809

[177] Fischer MT Sharma R Lim JL Haider L Frischer JM Drexhage J . NADPH oxidase expression in active multiple sclerosis lesions in relation to oxidative tissue damage and mitochondrial injury. Brain. (2012) 135:886–99. doi: 10.1093/brain/aws012 22366799 PMC3286337

[178] Koning N Swaab DF Hoek RM Huitinga I . Distribution of the immune inhibitory molecules CD200 and CD200R in the normal central nervous system and multiple sclerosis lesions suggests neuron-glia and glia-glia interactions. J Neuropathol Exp Neurol. (2009) 68:159–67. doi: 10.1097/nen.0b013e3181964113 19151626

[179] Traugott U Lebon P . Interferon-gamma and Ia antigen are present on astrocytes in active chronic multiple sclerosis lesions. J Neurol Sci. (1988) 84:257–64. doi: 10.1016/0165-5728(83)90036-x 3132537

[180] Vos CM van Haastert ES de Groot CJ van der Valk P de Vries HE . Matrix metalloproteinase-12 is expressed in phagocytotic macrophages in active multiple sclerosis lesions. J Neuroimmunol. (2003) 138:106–14. doi: 10.1016/s0165-5728(03)00036-5 12742660

[181] Ismail FS Hartung HP Melzer N . Anti-BCMA CAR-T cells attenuate microglial activation in progressive multiple sclerosis: Indicating a plasma cell-microglia crosstalk. Signal Transduct Target Ther. (2025) 10:407. doi: 10.1038/s41392-025-02510-6 41419461 PMC12717048

[182] Ortuño-Sahagún D Hermosillo-Abundis C Reyes-Mata MP Arias Carrión Ó . CAR T cells for multiple sclerosis: Engineering T cells to disrupt chronic B cell-driven neuroinflammation. Mult Scler Relat Disord. (2025) 104:106812. doi: 10.1016/j.msard.2025.106812 41176941

[183] Pecher AC Hensen L Schairer-Marquardt R Kowarik M Richter V Bethge W . Relapse after anti-CD19 CAR T-cell therapy in a patient with severe rheumatoid arthritis and multiple sclerosis effectively treated by autologous stem cell transplantation. Ann Rheum Dis. (2025) 84:1284–6. doi: 10.1016/j.ard.2025.03.018 40312206

[184] Yao Y Nguyen TD Pandya S Zhang Y Hurtado Rúa S Kovanlikaya I . Combining quantitative susceptibility mapping with automatic zero reference (QSM0) and myelin water fraction imaging to quantify iron-related myelin damage in chronic active MS lesions. AJNR Am J Neuroradiol. (2018) 39:303–10. doi: 10.3174/ajnr.a5482 29242359 PMC5812818

[185] KhorshidAhmad T Acosta C Cortes C Lakowski TM Gangadaran S Namaka M . Transcriptional regulation of brain-derived neurotrophic factor (BDNF) by methyl CpG binding protein 2 (MeCP2): A novel mechanism for re-myelination and/or myelin repair involved in the treatment of multiple sclerosis (MS). Mol Neurobiol. (2016) 53:1092–107. doi: 10.1007/s12035-014-9074-1 25579386

[186] McIntyre LL Greilach SA Othy S Sears-Kraxberger I Wi B Ayala-Angulo J . Regulatory T cells promote remyelination in the murine experimental autoimmune encephalomyelitis model of multiple sclerosis following human neural stem cell transplant. Neurobiol Dis. (2020) 140:104868. doi: 10.1016/j.nbd.2020.104868 32276110 PMC7253332

[187] Riboni-Verri G McMurran CE Mukherjee T Daruwalla C Holland J Gautam R . The Cambridge Centre for Myelin Repair trial Two (CCMR Two): A trial protocol for a phase 2a, randomised, double-blind, placebo-controlled clinical trial of the ability of the combination of metformin and clemastine to promote remyelination in people with relapsing-remitting multiple sclerosis already on disease-modifying therapy. Trials. (2025) 26:562. doi: 10.1186/s13063-025-09254-2 41361285 PMC12683890

[188] Barbuti E Conti A Treaba CA Miscioscia A Barletta VT Herranz E . Choroid plexus enlargement in multiple sclerosis correlates with cortical and phase rim lesions on 7T MRI and predicts progression independent of relapse activity. AJNR Am J Neuroradiol. (2026) 47:557–65. doi: 10.3174/ajnr.a8983 40854683 PMC12867058

[189] Rimkus CM Otsuka FS Nunes DM Chaim KT Otaduy MCG . Central vein sign and paramagnetic rim lesions: Susceptibility changes in brain tissues and their implications for the study of multiple sclerosis pathology. Diagnostics (Basel). (2024) 14. doi: 10.3390/diagnostics14131362 39001252 PMC11240827

[190] Gruber RC Wirak GS Blazier AS Lee L Dufault MR Hagan N . BTK regulates microglial function and neuroinflammation in human stem cell models and mouse models of multiple sclerosis. Nat Commun. (2024) 15:10116. doi: 10.1038/s41467-024-54430-8 39578444 PMC11584639

[191] Turner TJ Brun P Gruber RC Ofengeim D . Comparative CNS pharmacology of the Bruton's tyrosine kinase (BTK) inhibitor tolebrutinib versus other BTK inhibitor candidates for treating multiple sclerosis. Drugs R D. (2024) 24:263–74. doi: 10.1007/s40268-024-00468-4 38965189 PMC11315827

[192] Piancone F Saresella M Marventano I La Rosa F Zoppis M Agostini S . B lymphocytes in multiple sclerosis: Bregs and BTLA/CD272 expressing-CD19+ lymphocytes modulate disease severity. Sci Rep. (2016) 6:29699. doi: 10.1038/srep29699 27412504 PMC4944189

[193] Radmilo M Pavelin S Vujović I Šoda J Rogić Vidaković M . Confirmation of CD19+ B-lymphocyte depletion prior to intake of the second dose of ocrelizumab in multiple sclerosis patients. Biomedicines. (2023) 11. doi: 10.3390/biomedicines11020353 36830890 PMC9953738

[194] Borggrewe M Kooistra SM Wesseling EM Gierschek FL Brummer ML Nowak EC . VISTA regulates microglia homeostasis and myelin phagocytosis, and is associated with MS lesion pathology. Acta Neuropathol Commun. (2021) 9:91. doi: 10.1186/s40478-021-01186-7 34006329 PMC8130385

[195] Cignarella F Filipello F Bollman B Cantoni C Locca A Mikesell R . TREM2 activation on microglia promotes myelin debris clearance and remyelination in a model of multiple sclerosis. Acta Neuropathol. (2020) 140:513–34. doi: 10.1007/s00401-020-02193-z 32772264 PMC7498497

[196] Fani Maleki A Rivest S . Innate immune cells: Monocytes, monocyte-derived macrophages and microglia as therapeutic targets for Alzheimer's disease and multiple sclerosis. Front Cell Neurosci. (2019) 13:355. doi: 10.3389/fncel.2019.00355 31427930 PMC6690269

[197] Kirschbaum K Sonner JK Zeller MW Deumelandt K Bode J Sharma R . *In vivo* nanoparticle imaging of innate immune cells can serve as a marker of disease severity in a model of multiple sclerosis. Proc Natl Acad Sci USA. (2016) 113:13227–32. doi: 10.1073/pnas.1609397113 27799546 PMC5135308

[198] Hametner S Dal Bianco A Trattnig S Lassmann H . Iron related changes in MS lesions and their validity to characterize MS lesion types and dynamics with ultra-high field magnetic resonance imaging. Brain Pathol. (2018) 28:743–9. doi: 10.1111/bpa.12643 30020556 PMC8028547

[199] Baudron E Martinez de Lizarrondo S Gauberti M Delaunay-Piednoir B Fournier AP Vivien D . Intestinal MAdCAM-1 imaging as biomarker for prognostic in murine models of multiple sclerosis. Brain Behav Immun. (2024) 119:381–93. doi: 10.1016/j.bbi.2024.04.006 38604270

[200] Malinick AS Lambert AS Stuart DD Li B Puente E Cheng Q . Detection of multiple sclerosis biomarkers in serum by ganglioside microarrays and surface plasmon resonance imaging. ACS Sens. (2020) 5:3617–26. doi: 10.1021/acssensors.0c01935 33115236 PMC8371921

[201] Miscioscia A Mainero C Treaba CA Silvestri E Scialpi G Berardi A . The contribution of paramagnetic rim and cortical lesions to physical and cognitive disability at multiple sclerosis clinical onset: evaluating the power of MRI and OCT biomarkers. J Neurol. (2024) 271:6702–14. doi: 10.1007/s00415-024-12622-8 39155316

[202] De Kleijn KMA Martens GJM . Molecular effects of FDA-approved multiple sclerosis drugs on glial cells and neurons of the central nervous system. Int J Mol Sci. (2020) 21. doi: 10.3390/ijms21124229 32545828 PMC7352301

[203] Yenikekaluva A Sjöros T Matilainen M Snellman A Rinne J Alfaifi BQ . TSPO-PET-measurable neuroinflammation associates with brain atrophy in multiple sclerosis. Mult Scler Relat Disord. (2026) 110:107207. doi: 10.1016/j.msard.2026.107207 42025273

